# Sex/Gender Differences in Verbal Fluency and Verbal-Episodic Memory: A Meta-Analysis

**DOI:** 10.1177/17456916221082116

**Published:** 2022-07-22

**Authors:** Marco Hirnstein, Josephine Stuebs, Angelica Moè, Markus Hausmann

**Affiliations:** 1 Department of Biological and Medical Psychology, University of Bergen; 2 Department of Neuropsychology and Psychopharmacology, Maastricht University; 3 Institute of Clinical Medicine, University of Oslo; 4 Department of General Psychology, University of Padua; 5 Department of Psychology, Durham University

**Keywords:** verbal ability, phonemic fluency, semantic fluency, verbal memory, age, author effects

## Abstract

Women are thought to fare better in verbal abilities, especially in verbal-fluency and verbal-memory tasks. However, the last meta-analysis on sex/gender differences in verbal fluency dates from 1988. Although verbal memory has only recently been investigated meta-analytically, a comprehensive meta-analysis is lacking that focuses on verbal memory as it is typically assessed, for example, in neuropsychological settings. On the basis of 496 effect sizes and 355,173 participants, in the current meta-analysis, we found that women/girls outperformed men/boys in phonemic fluency (*d*s = 0.12–0.13) but not in semantic fluency (*d*s = 0.01–0.02), for which the sex/gender difference appeared to be category-dependent. Women/girls also outperformed men/boys in recall (*d* = 0.28) and recognition (*d*s = 0.12–0.17). Although effect sizes are small, the female advantage was relatively stable over the past 50 years and across lifetime. Published articles reported stronger female advantages than unpublished studies, and first authors reported better performance for members of their own sex/gender. We conclude that a small female advantage in phonemic fluency, recall, and recognition exists and is partly subject to publication bias. Considerable variance suggests further contributing factors, such as participants’ language and country/region.

After more than 100 years of psychological research, sex/gender^1^ differences in cognitive abilities are still heavily debated (for reviews, see Halpern, 2012; Hyde, 2014). Spatial and mathematical abilities, in which men are commonly believed to excel, are very well researched. For instance, a male advantage in mental rotation, the ability to rotate complex figures in one’s mind, has been reported in several meta-analyses with effect sizes around Cohen’s *d* from 0.56 to 0.73 (Linn & Petersen, 1985; Voyer et al., 1995; Zell et al., 2015). By comparison, much less is known about verbal abilities, in which women/girls are commonly believed to excel. There is no unitary concept of verbal abilities, but it relates to all aspects of open or inner language production and comprehension. Meta-analyses reported female advantages with medium effect sizes for writing ability (*d*s = 0.53–0.61; Hedges & Nowell, 1995) and reading comprehension (*d*s = 0.23–0.68; Reilly, 2012; Stoet & Geary, 2013). Verbal intelligence/reasoning (Feingold, 1988) and vocabulary (Hyde & Linn, 1988), on the other hand, did not reveal a female advantage (effect sizes smaller than *d* = 0.05; Hyde, 2005, 2014).

The two verbal abilities, however, that textbooks and review articles typically refer to when claiming the existence of a female advantage are verbal fluency (sometimes also called “word fluency”) and verbal memory (Andreano & Cahill, 2009; Halpern, 2012; Hamson et al., 2016; Hyde, 2014; Kimura, 2000; Miller & Halpern, 2014). Verbal-fluency and verbal-memory tests correlate with general cognitive abilities (Alexander & Smales, 1997; Kraan et al., 2013) and are frequently used in psychological assessments of developmental impairments in children (Gaillard et al., 2003; Pennington & Ozonoff, 1996), impairments and rehabilitation after stroke (Baldo et al., 2006; Barker-Collo & Feigin, 2006), and cognitive decline in dementia (Collie & Maruff, 2000; Zhao et al., 2013).

## Verbal Fluency

Verbal fluency refers to the ability to generate (orally or written) as many words as possible that fulfill a certain criterion, normally under time restrictions. The criterion is typically either semantic, also called “categorical fluency” (e.g., naming animals, fruits, etc.) or phonemic (e.g., naming words that begin with a specific letter), also called “lexical/letter fluency.” Virtually all articles that claim women’s/girls’ superiority in verbal fluency refer to a landmark meta-analysis by Hyde and Linn (1988), who examined sex/gender differences in a few verbal abilities. The authors concluded that “speech production” or “verbal production” favored women by *d* = 0.33. However, the definition of “speech production” (“as occurs in essay writing or measures of spoken language,” p. 55) is different from the verbal-fluency definition above, and consequently, some studies in Hyde and Linn (1988) assessed different verbal abilities, such as quality of essays or written sentences (Harris & Seibel, 1976; Wormack, 1979) or how many words 4-year-old children speak (Brownell & Smith, 1973). Moreover, the meta-analysis was based on only 14 studies, whereas the Web of Knowledge revealed that approximately 7,500 references have included the term “verbal fluency” since 1988.

## Phonemic Versus Semantic Fluency, Age, Cohort Effects, and Gender of First/Last Author

Heister (1982) found a female advantage when participants were asked to generate words beginning with the letters “S” and “M” (phonemic fluency), whereas no sex/gender differences emerged for naming things that are red or round (semantic fluency). Other studies reported a female advantage in semantic fluency (Acevedo et al., 2000) or did not find a sex/gender difference in either phonemic or semantic fluency (Kavé, 2005). Overall, it is unclear whether a female advantage exists in both semantic and phonemic fluency.

Furthermore, it is unclear at what age the putative female advantage arises and whether it changes across the life span. Some studies suggest a steeper decline in older men compared with women (Maylor et al., 2007; Rodriguez-Aranda & Martinussen, 2006), whereas de Frias et al. (2006) found that the female advantage in semantic fluency was stable between 35 and 80 years. On the basis of semantic fluency data from more than 30,000 individuals (ages 50–84) in 14 European countries, Weber et al. (2014, 2017) showed that women from younger cohorts performed better than women from older cohorts. Sex/gender differences also varied across European countries. Both findings were interpreted to show the impact of better access of women to resources and education (Weber et al., 2014, 2017). So far, it is unclear whether sex/gender differences in verbal fluency change with age or across cohorts.

Finally, Hyde and Linn (1988) found that female first authors reported a stronger female advantage (*d* = 0.15) than male first authors (*d* = 0.08). However, this finding was based on all verbal abilities, and although statistically significant, the difference was considered to be unsubstantial. In the current study, we sought to replicate the findings by Hyde and Linn but more specifically with respect to verbal fluency. In addition, we also investigated the influence of gender of the last author, who is often the supervisor or more senior researcher overseeing the research effort.

## Verbal-Episodic Memory

As with verbal ability, there is no unitary definition of verbal memory. Nevertheless, there is a multitude of empirical data on what researchers considered verbal memory. Several studies found better performance in women (Catani et al., 2007; de Frias et al., 2006; Herlitz et al., 1997; P. A. Lowe et al., 2003), and a narrative review concluded that “females show an advantage at verbal memory” (Andreano & Cahill, 2009, p. 260). However, other studies found no sex/gender differences in verbal memory (Munnelly, 2016; Parsons et al., 2005). Meta-analyses on this issue were lacking until recently. Voyer et al. (2021) focused specifically on verbal working memory and found an overall significant female advantage that, however, was practically zero (Hedge’s *g* = 0.03). Furthermore, sex/gender differences varied across different sample and task parameters: Tasks with cued recall (*g* = 0.08) and free recall (*g* = 0.15) had a slightly elevated female advantage, whereas there was a male advantage in complex span (*g* = 0.04) and no significant sex/gender difference in serial recall (*g* < 0.01) and simple span (*g* < 0.01).

Another meta-analysis (Asperholm et al., 2019) investigated sex/gender differences in long-term memory, specifically episodic memory. Long-term memory is typically divided into declarative (explicit) and nondeclarative (implicit) memory; declarative memory comprises episodic memory (i.e., the ability to remember specific events or situations at a particular place at a particular time) and semantic memory (i.e., the ability to remember concepts and facts). Asperholm et al. (2019) investigated sex/gender differences in episodic memory for different stimuli, including images, movies, faces, routes, locations, and verbal content such as words/sentences. Verbal content showed a small female advantage (*g* = 0.28). A wide range of studies/tasks were included in the verbal-episodic category, and the authors investigated whether the female advantage varied across, for example, neutral stimuli versus emotional stimuli, intentionally learned versus incidentally learned, or recall versus recognition. Subsequent analyses of moderator variables, such as age, publication year, or geographical region, took into account whether the stimulus material was verbal, images, movies, or faces but did not distinguish between incidental/intentional, emotional/neutral, or recall/recognition, and only peer-reviewed articles were included.

Like Asperholm et al. (2019), in the present study, we were interested in episodic long-term memory and thus discarded studies/tasks that primarily assess working memory. In contrast to Asperholm et al., we had a narrower focus on verbal-episodic memory, which we investigated with a broader literature search. That is, we examined exclusively verbal-episodic memory (not memory for routes and locations) and included only studies with neutral stimuli (vs. emotional stimuli) in which participants learned material intentionally (vs. incidentally). The intentional learning of neutral stimuli is a key feature of frequently used neuropsychological tests on verbal long-term memory, such as the California Verbal Learning Test (CVLT; Delis et al., 2000), the Rey Auditory Verbal Learning Test (RAVLT; Schmidt, 1996), or the Wechsler Memory Scale (WMS; Wechsler, 2009). Further in contrast to Asperholm et al., the literature search of the current study also included “gray” literature, such as PhD/master’s theses, to investigate whether sex/gender differences are subject to publication effects. Moreover, the current study examined, for the first time, possible effects of first/last authors’ gender on sex/gender differences in verbal-episodic memory. Finally, we performed these analyses separately for recognition (i.e., when cues are provided for the material that had to be memorized) and recall (i.e., absence or lack of cues) because the female advantage appeared to be consistently larger for recall than for recognition (Asperholm et al., 2019; Voyer et al., 2021). The fact that only 14 and 18 of our 168 included studies overlapped with Voyer et al. (2021) and Asperholm et al., respectively, demonstrates that different aspects of verbal memory were investigated in the current study. Henceforth, we thus use the term “verbal-episodic memory” to refer to the data that were analyzed in the present study and “verbal memory” to refer to verbal memory in general.

## Aims and Hypotheses

A female advantage is frequently assumed in verbal fluency and verbal memory. For verbal fluency, this assumption is based on an early meta-analysis by Hyde and Linn (1988) that required an update. For verbal memory, a meta-analysis was missing that focuses specifically on verbal-episodic memory—complementary to two recent meta-analyses about verbal working memory (Voyer et al., 2021) and episodic memory in general (Asperholm et al., 2019). In the present study, we thus aimed to reveal the magnitude of the putative female advantage in verbal fluency and verbal-episodic memory. For both, we additionally examined the impact of potentially modulating factors such as publication year, type of publication (articles vs. PhD/master theses), participants’ age, semantic fluency versus phonemic fluency, recall versus recognition, and gender of first/last author. We hypothesized a female advantage (a) in both verbal fluency and verbal-episodic memory of intentionally learned neutral stimuli (Andreano & Cahill, 2009; Halpern, 2012; Miller & Halpern, 2014), (b) that has increased over the past 50 to 60 years because of better access to education for women (Weber et al., 2014, 2017), (c) that emerges across all age groups but becomes larger in older adults (Maylor et al., 2007; Rodriguez-Aranda & Martinussen, 2006), and (d) that is affected by the gender of the first (Hyde and Linn, 1988) and last authors.

## Method

The meta-analysis, including literature search, study selection, data analysis, and presentation of results, was performed following the PRISMA (Preferred Reporting Items for Systematic Reviews and Meta-Analyses) guidelines (Moher et al., 2009) and the recommendations for meta-analyses described by Borenstein et al. (2009). Data analysis was carried out with Comprehensive Meta-Analysis (Version 3.3.070; Borenstein et al., 2014).

### Literature search and study selection

#### Search terms and databases

Between October 22 and 29, 2016, the databases PsychInfo, ISI Web of Knowledge, and PubMed were searched for relevant literature. Between September 13 and 19, 2019, we additionally searched the ProQuest Dissertation & Theses database to identify unpublished PhD and master’s theses. For the search terms and number of identified references, see Table S1 in the Supplemental Material available online. An additional 16 studies were identified through other sources, such as comprehensive literature reviews and references used in previously identified publications. After removing 38,322 duplicates, the remaining 28,305 hits were screened for suitability. Screening comprised reading both title and full abstract. In isolated cases, references were excluded based solely on title, for example, in case the title indicated that the reference was a review or meta-analysis without original data or the topic of the reference was outside the scope of the present meta-analysis (e.g., “Persephone in the Underworld: The Motherless Hero in Novels by Burney, Radcliffe, Austen, Bronte, Eliot, and Woolf”). Some older PhD and master’s theses often did not have abstracts, in which case the whole thesis was screened. Details about the exclusion criteria and procedure during screening is provided in the Supplemental Material.

#### Study selection: final inclusion criteria

Of the 2,984 references that were included after screening of abstract/title, 72 full texts could not be obtained. The remaining 2,912 references then underwent a full-text search for eligibility. Inclusion criteria were:

1. Use of phonemic/semantic-fluency and/or verbal-episodic-memory (recognition/recall) tests that comply with the aforementioned definitions of verbal fluency and verbal-episodic memory. Examples for verbal fluency are the Controlled Oral Word Association Test (COWAT; Benton, 1967) or the F-A-S Test (Spreen & Benton, 1977), the Thurstone Word Fluency Test (Thurstone & Thurstone, 1962), or any test in which participants had to generate as many words as possible starting/ending with or containing certain letters and to provide as many examples as possible for a specific category. Not included were data from tests such as finding synonyms or essay writing (which were considered too peripheral for verbal fluency). Anagram tasks were excluded on the grounds that they draw on numerical and spatial abilities (Wilson et al., 1954).
  For verbal-episodic memory, we excluded tasks that measured exclusively or predominantly working memory such as digit span forward or backward from the Wechsler Adult Intelligence Scales (Wechsler, 2008). Examples for included verbal-episodic memory tests are the Visual Verbal Learning Test (Brand & Jolles, 1985), the RAVLT, and the CVLT. Logical Memory II and Logical Memory Recognition (remembering a story) from the WMS were included, but not Logical Memory I because this subtest is more related to verbal working memory. If multiple verbal-episodic-memory parameters were provided (e.g., delayed recall, total recall, recall), we retained the total score; otherwise, the provided scores were kept. Learning in all verbal-episodic-memory measures had to be intentional (i.e., incidental learning measures were not included).
2. For both verbal fluency and episodic memory, we excluded tasks that employed emotional stimuli because they could be confounded with sex/gender differences in emotional processing (Kret & De Gelder, 2012; Stevens & Hamann, 2012). For example, affective semantic-fluency categories such as “pleasant/unpleasant” or “joy/fear” (e.g., Gawda & Szepietowska, 2013a, 2013b) were not included.
3. Verbal-fluency/episodic-memory stimuli were not presented laterally, that is, to one specific hemisphere. For example, tasks that employed laterality paradigms were not considered because of sex/gender differences in hemispheric asymmetry (Hirnstein et al., 2019).
4. Verbal-fluency/episodic-memory tasks were not performed simultaneously with other tasks because multitasking abilities might vary across men and women (Hirnstein et al., 2018).
5. The publication contained quantitative, empirical data (i.e., no reviews, study protocols, meta-analyses), which allowed computation of the effect size and the exact number (or percentages) of male and female participants. Only “pure” verbal-fluency and verbal-episodic-memory measures were included. That is, if covariates such as intelligence had been factored in, the data were excluded. If only aggregate scores were provided from test batteries that included both eligible and not eligible tasks, data were excluded. Finally, when studies reported multiple verbal-fluency/episodic-memory tasks but provided only statistical parameters to compute effect sizes for tests that found significant sex/gender differences—and insufficient statistical parameters for tests that did not find sex/gender differences—the whole study was discarded to avoid introducing a bias toward significant results.
6. There were at least 10 male and 10 female participants in the sample to mitigate the effect of spurious findings with very small sample sizes.
7. Participants were healthy individuals without a mental or other condition that could affect verbal-fluency/episodic-memory performance (e.g., depression, Alzheimer’s disease, learning disability) and were not under the influence of any kind of substance, medicine, or other factors that might influence cognitive performance (e.g., sleep deprivation, noise exposure). Data from control groups could be included unless control subjects were selected for specific features (e.g., intelligence, age, socioeconomic status) to match clinical groups.
8. Participants were not preselected for a specific feature that could potentially be related to verbal-fluency/episodic-memory performance (e.g., participants with certain gene combination or combinations, participants who performed better than average on a creativity test, samples with homosexual participants only).
9. The publication was written in English, German, or any Scandinavian language.
10. Cohen’s *d* was outside the range of −4.0 to 4.0, which we deemed unrealistic. The range of included effect sizes was −1.07 to 1.42.

For cases in which inclusion criteria were met but the study lacked important quantitative information (e.g., number of men/women/boys/girls, means, or *p* values), authors were contacted with a request to provide the relevant data and other relevant data they have or know of. Out of 45 contacted authors, nine provided relevant data.

**Fig. 1.**
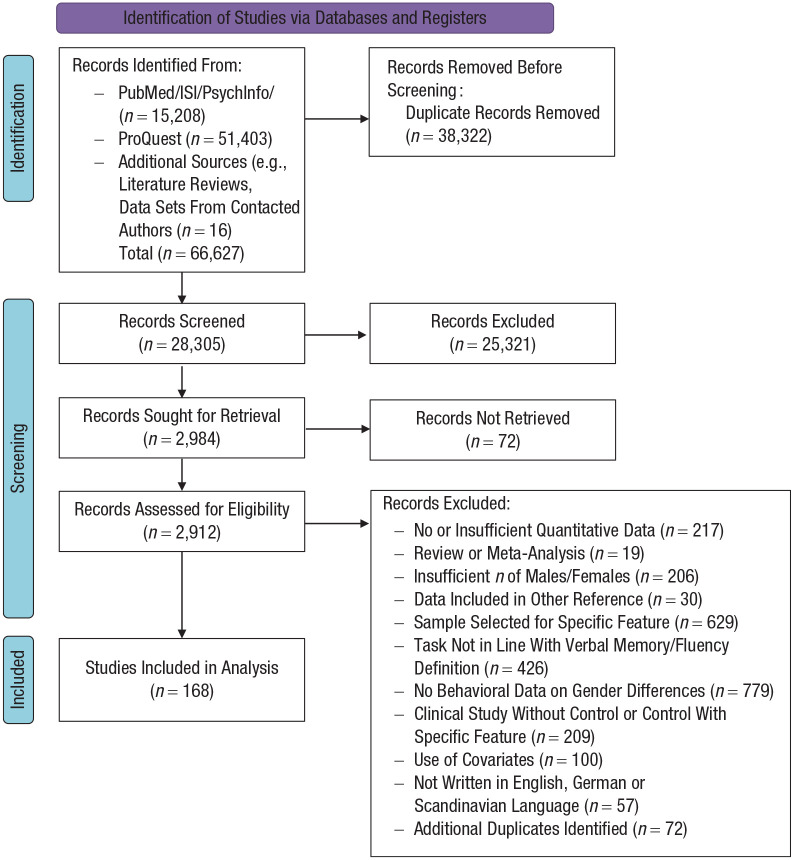
PRISMA (Preferred Reporting Items for Systematic Reviews and Meta-Analyses) flow diagram showing the study-selection process.

In total, 496 effect sizes from 168 references were included for quantitative analysis, comprising data from 355,173 participants (men/boys = 178,409, women/girls = 176,764). For a more detailed overview of the study-selection process, including reasons that led to exclusion, see Figure 1. For a complete list of all included references and effect sizes, see Table S2 in the Supplemental Material.

### Statistical analysis

For each relevant measure from the included references above, standardized differences in means (Cohen’s *d*) were computed from the available statistical information. If the male/female distribution was given in percentages, they were converted into integers. The effect direction was set such that positively signed values indicate a female advantage and negatively signed values indicate a male advantage. A value of zero indicates the absence of any male/female advantage. We consistently applied the random-effects model because (a) we expected substantial between-studies variance and (b) we aimed to generalize our findings to the entire population. Moreover, we consistently used subgroups in a reference as the unit of analysis (vs. using the whole reference as the unit of analysis). That is, if a study included a verbal-episodic-memory measure from two age groups (e.g., one 50–59 and another 60–69), those subgroups were treated as separate measures rather than combining them into one measure.

Several studies reported multiple outcomes for each sample/subsample. For example, a study could provide data from two different tests that both measure recall. It is likely that those tests were correlated with each other and that the magnitude of that correlation affects the variance and, thus, the likelihood of finding statistically significant results (Borenstein et al., 2009). Because these correlations were rarely reported, we ran each analysis twice: once with *r* = 0, assuming perfect independence of the outcomes, and once with *r* = 1.0, assuming perfect correlation between outcomes. In most cases, the results of both analyses yielded similar results. For ease of reading, we always report the perfect independence results first. All tables/figures were based on the assumption of perfect independence.

#### Overall sex/gender effects

First, we computed the overall sex/gender effect separately for verbal fluency and verbal-episodic memory. Then, we computed the overall sex/gender effect for each of the following four verbal-ability measures: phonemic and semantic fluency as measures of verbal fluency and recognition and recall as measures of verbal-episodic memory. One study had aggregated phonemic- and semantic-fluency scores into a combined verbal-fluency score (DeWan, 2006), whereas another had aggregated recognition and recall scores into combined verbal-episodic-memory scores (Rouch et al., 2005). Effect sizes from these studies were thus kept in the overall verbal-fluency/episodic-memory analysis but excluded from the recognition/recall/phonemic/semantic-fluency analysis.

For all these analyses, we provide *Q* statistic (testing the null hypothesis that all studies in the analysis shared a common effect size), *I*^2^ (the proportion of observed variance that reflects difference in true effect sizes rather than sampling error), and *T*^2^ (the variance of true effect sizes) as indicators of how much the sex/gender effect varied across studies. To address the issue of publication bias, we reported Egger’s regression (two-tailed; Egger et al., 1997) and funnel plots (see Fig. S1 in the Supplemental Material).

#### Effects of publication year, publication type, age, and gender of first/last authors

To investigate whether sex/gender differences change with publication year (as an indicator for changes over time), vary across publication type (articles vs. PhD/master’s theses), age, and the gender of the first/last authors, we ran a set of metaregressions. Metaregressions have the advantage that they allow investigating the effect of one factor while controlling for a set of other factors (Borenstein et al., 2009). Here again, we assumed that the true effect size varied across studies and thus applied a random-effects model (method of moments). All tests were two-sided and based on *z* distribution.

Six covariates were created for the metaregressions: (a) The continuous covariate “publication year” simply coded the year when a reference was published. (b) “Publication type” was a categorical covariate that could either be “published article” or “PhD/master’s thesis.” (c) Age was analyzed with two covariates: “mean age” as a continuous variable, which was either obtained directly from the corresponding reference or, in case that information was missing, computed on the basis of the age range (e.g., an age range of 40–60 would lead to a mean age of 50). If age ranges were provided separately for men/boys and women/girls, we took the youngest and oldest age from either sex/gender. If mean ages were provided separately for women/girls and men/boys, we calculated a weighted overall mean. Using mean age alone, however, has two shortcomings. First, several studies provided only age information such as “>70 years,” which made it impossible to calculate a mean. Second, many studies have enormous age ranges. For example, approximately 20% of studies had age ranges of 40 years and more, which rendered mean age a rather coarse indicator. (d) For this reason, we created a second covariate to examine age effects: “age groups.” This was a categorical covariate, theoretically grounded in the Medical Subject Heading, the standardized vocabulary used in the Medline database for indexing, developed by National Library of Medicine. According to this classification, the following age categories were formed: “child/child preschool” (2–12), “adolescent” (13–18), “adult” (19–44), “middle aged” (45–64), and “aged” (65+). Effect sizes were grouped into those categories using the reported age range of the corresponding study. For example, an effect size based on a sample with an age range of 20 to 27 was classified as adult. An effect size based on an age range of 17 to 40 was coded blank and excluded from the age-groups analysis. As a consequence, the number of effect sizes was substantially higher for mean age (92%, 455/497) than for age groups (51%, 253/497). Although both age measures have their respective shortcomings, we combined both because this allows a reasonable estimate of age effects (see also Voyer et al., 2021). Finally, (e) and (f) were the categorical covariates “first author gender” and “last author gender,” respectively, which was either male or female. In case of single-author studies, this was coded as first author and was not included for analysis of last-author effects.

The categorical covariates described above were dummy-coded in order to be entered into the metaregression. This was done such that published articles, males, and adult served as reference groups for publication type, first/last author gender, and age groups, respectively. We did not include language as a covariate because there were too few non-English reports of data. For comparison, 263 out of 496 effect sizes (53%) were reported in English, whereas the second most frequent language, Dutch, comprised only 40 effect sizes (8%).

We ran a sequence of metaregressions for each verbal ability (i.e., recall, recognition, phonemic/semantic fluency) separately. The first metaregression always included the covariates publication year, mean age, publication type, and first-author gender. This was done to maximize the number of available effect sizes. Age groups was not entered into the first metaregression because of multicollinearity with mean age and because only half of the effect sizes could be assigned to a specific age group (see above). We thus ran a second metaregression that included age group and all significant covariates from the first metaregression as a control (except for mean age because of multicollinearity). Last-author gender was also not entered into the first metaregression because of multicollinearity with publication type: None of the PhD/master’s theses have a last author. Therefore, we ran a third metaregression for published articles that included only last-author gender and all significant covariates from the first metaregression as a control (except for publication type because of multicollinearity).

## Results

### Overall sex/gender differences

Effect sizes of the most frequent verbal-fluency and verbal-episodic-memory measures are presented in Table 1.

**Table 1.** Descriptive Overview of Sex/Gender Differences in Verbal-Fluency and Verbal-Episodic-Memory Measures

Verbal ability	Test/measure	Effect size
Verbal fluency	Total effect	*d* = 0.07 [0.04, 0.10], *k* = 290
Phonemic fluency	Total effect	*d* = 0.13 [0.09, 0.16], *k* = 135
	Generic starting letter(s)	*d* = 0.12 [0.07, 0.18], *k* = 59
	Controlled Oral Word Association Test/F-A-S Test	*d* = 0.14 [0.08, 0.20], *k* = 55
	Four-word sentences	*d* = 0.03 [−0.20, 0.26], *k* = 5
Semantic fluency	Total effect	*d* = 0.02 [−0.02, 0.06], *k* = 147
	Category: animals	*d* = −0.13 [−0.16, −0.09], *k* = 58
	Categories: animals and fruits/vegetables/food	*d* = 0.11 [0.03, 0.18], *k* = 26
	Objects with specific color	*d* = 0.19 [0.13, 0.25], *k* = 10
	Categories: animals, fruits/vegetables/food, and action verbs	*d* = 0.25 [−0.03, 0.53], *k* = 8
	Fruits/vegetables/food	*d* = 0.31 [0.16, 0.47], *k* = 8
Verbal-episodic memory	Total effect	*d* = 0.23 [0.19, 0.26], *k* = 206
Recall	Total effect	*d* = 0.28 [0.23, 0.32], *k* = 136
	California Verbal Learning Test	*d* = 0.42 [0.32, 0.52], *k* = 28
	Rey Auditory Verbal Learning Test	*d* = 0.39 [0.29, 0.48], *k* = 24
	Generic word list	*d* = 0.17 [0.06, 0.28], *k* = 16
	Delayed Memory for Names/Visual-Auditory Learning from Woodcock Johnson Psycho-Educational Battery–Revised	*d* = −0.13 [−0.27, 0.01], *k* = 12
	10 Word Learning Test from CERAD	*d* = 0.18 [0.07, 0.28], *k* = 10
	Ten-Words Test	*d* = 0.26 [0.13, 0.39], *k* = 7
	Deese, Roediger, and McDermott task	*d* = 0.15 [0.02, 0.28], *k* = 7
Recognition	Total effect	*d* = 0.12 [0.06, 0.17], *k* = 66
	Rey Auditory Verbal Learning Test	*d* = 0.22 [0.12, 0.33], *k* = 18
	California Verbal Learning Test	*d* = 0.17 [0.06, 0.29], *k* = 13
	Deese, Roediger, and McDermott task	*d* = 0.15 [0.04, 0.27], *k* = 7
	Storytelling delayed recognition	*d* = −0.07 [−0.18, 0.04], *k* = 7
	Storytelling immediate recognition	*d* = 0.02 [−0.09, 0.13], *k* = 7

Note: Values in brackets represent 95% confident intervals; *k* = number of effect sizes included. Effect sizes are provided assuming independence between multiple outcomes in the same study. Effect sizes in each subcategory were combined with a random-effects model, assuming a common among-study variance component across subcategories. That is, *T*^2^ was computed for each age group and then pooled across subgroups. Only tests with at least seven effect sizes are provided, except for phonemic fluency, for which the three most frequent tests are provided. CERAD = Consortium to Establish a Registry for Alzheimer’s Disease.

#### Verbal fluency

Assuming perfect independence between multiple outcomes in the same study, we found that the overall effect size was *d* = 0.07 with a 95% confidence interval (CI) of 0.04 to 0.10, based on 290 effect sizes. The female advantage deviated significantly from zero, *Z* = 5.10, *p* < .001. There was substantial heterogeneity among studies, *Q*(289) = 2085.1, *p* < .001, *I*^2^ = 86.1%, *T*^2^ = 0.02. Egger’s regression intercept of −0.10 was not significant, *t*(288) = 0.54, *p* = .591.

Assuming perfect correlation between multiple outcomes in the same study, we found that all effects remained significant/nonsignificant: *d* = 0.07, 95% CI = [0.04, 0.10], *Z* = 4.60, *p* < .001, *Q*(209) = 1784.3, *p* < .001, *I*^2^ = 88.3%, *T*^2^ = 0.02, Egger’s intercept = −0.13, *t*(208) = 0.52. *p* = .602, based on 210 effect sizes.

#### Verbal-episodic memory

Assuming perfect independence, we found a significant female advantage, *d* = 0.23, 95% CI = [0.19, 0.26], *Z* = 13.09, *p* < .001, based on 206 effect sizes. Heterogeneity was substantial, *Q*(205) = 1622.7, *p* < .001, *I*^2^ = 87.4%, *T*^2^ = 0.04. Egger’s intercept was 1.08, *t*(204) = 3.94, *p* < .001. Assuming perfect correlation, we found that all effects remained significant/nonsignificant: *d* = 0.26, 95% CI = [0.21, 0.30], *Z* = 11.39, *p* < .001, *Q*(132) = 1194.1, *p* < .001, *I*^2^ = 88.9%, *T*^2^ = 0.04, Egger’s intercept = 1.18, *t*(131) = 3.45, *p* < .001, based on 133 effect sizes.

#### Phonemic fluency

There was a significant female advantage, *d* = 0.13, 95% CI = [0.09, 0.16], *Z* = 6.75, *p* < .001, based on 135 effect sizes. There was significant heterogeneity, *Q*(134) = 272.3, *p* < .001, *I*^2^ = 50.8%, *T*^2^ = 0.01. Egger’s intercept was 0.19, *t*(133) = 1.04, *p* = .30. Assuming perfect correlation, we found that all effects remained significant/nonsignificant: *d* = 0.12, 95% CI = [0.09 0.16], *Z* = 6.97, *p* < .001, *Q*(128) = 226.9, *p* < .001, *I*^2^ = 43.6%, *T*^2^ = 0.01, Egger’s intercept = 0.20, *t*(127) = 1.14. *p* = .25, based on 129 effect sizes.

#### Semantic fluency

There was no significant sex/gender difference in semantic fluency, *d* = 0.02, 95% CI = [−0.02 0.06], *Z* = 1.00, *p =* .315, based on 147 effect sizes. The effect varied significantly across studies, *Q*(146) = 1782.6, *p* < .001, *I*^ 2^ = 91.8%, *T*^ 2^ = 0.03, and Egger’s intercept was −0.61, *t*(145) = 1.78, *p* = .078. Assuming perfect correlation, we found that all effects remained significant/nonsignificant: *d* = 0.01, 95% CI = [−0.02 0.05], *Z* = 0.70, *p* = .482, *Q*(136) = 1740.1, *p* < .001, *I*^ 2^ = 92.2%, *T*^ 2^ = 0.03, Egger’s intercept = −0.68, *t*(135) = 1.86. *p* = .065, based on 137 effect sizes.

#### Recall

There was a significant female advantage, *d* = 0.28, 95% CI = [0.23, 0.32], *Z* = 12.54, *p <* .001, based on 136 effect sizes. The effect varied largely between studies, *Q*(135) = 1217.0, *p* < .001, *I*^ 2^ = 88.9%, *T*^ 2^ = 0.04. Egger’s intercept was 1.32, *t*(134) = 3.94, *p* < .001. Assuming perfect correlation, we found that all effects remained significant/nonsignificant: *d* = 0.28, 95% CI = [0.24, 0.33], *Z* = 11.90, *p* < .001, *Q*(123) = 1155.3, *p* < .001, *I*^ 2^ = 89.4%, *T*^ 2^ = 0.04, Egger’s intercept = 1.35, *t*(123) = 3.85. *p* < .001, based on 124 effect sizes.

#### Recognition

There was a significant female advantage, *d* = 0.12, 95% CI = [0.06 0.17], *Z* = 4.42, *p <* .001, 66 effect sizes. The effect varied significantly across studies, *Q*(65) = 257.1, *p* < .001, *I*^ 2^ = 74.7%, *T*^ 2^ = 0.02. Egger’s intercept was 1.27, *t*(64) = 3.11, *p* = .003. Assuming perfect correlation, we found that all effects remained significant/nonsignificant: *d* = 0.17, 95% CI = [0.10, 0.24], *Z* = 4.78, *p* < .001, *Q*(49) = 164.9, *p* < .001, *I*^ 2^ = 70.3%, *T*^ 2^ = 0.03, Egger’s intercept = 1.08, *t*(48) = 2.42. *p* = .019, based on 50 effect sizes.

### Metaregressions for moderator variables

The first set of metaregressions contained the predictors publication year, publication type, first-author gender, and mean age. Assuming perfect independence, we found that all four models explained a significant proportion of between-studies variance: phonemic fluency, *Q*(4) = 15.75, *p* = .003, *R*^2^ = 3.6, based on 125 effect sizes; semantic fluency, *Q*(4) = 28.94, *p* < .001, *R*^2^ = 51.0%, based on 129 effect sizes; recall, *Q*(4) = 28.76, *p* < .001, *R*^2^ = 23.5%, based on 124 effect sizes; and recognition, *Q*(4) = 33.03, *p* < .001, *R*^2^ = 31.3%, based on 65 effect sizes. Assuming perfect correlation, we found that all four models remained significant: phonemic fluency, *Q*(4) = 18.04, *p* = .001, *R*^2^ = 11.2%, based on 119 effect sizes; semantic fluency, *Q*(4) = 35.66, *p* < .001, *R*^2^ = 53.2, based on 120 effect sizes; recall, *Q*(4) = 25.89, *p* < .001, *R*^2^ = 23.9, based on 111 effect sizes; and recognition, *Q*(4) = 23. 80, *p* < .001, *R*^2^ = 36.2, based on 49 effect sizes.

#### Published articles versus PhD/master’s theses

**Fig. 2.**
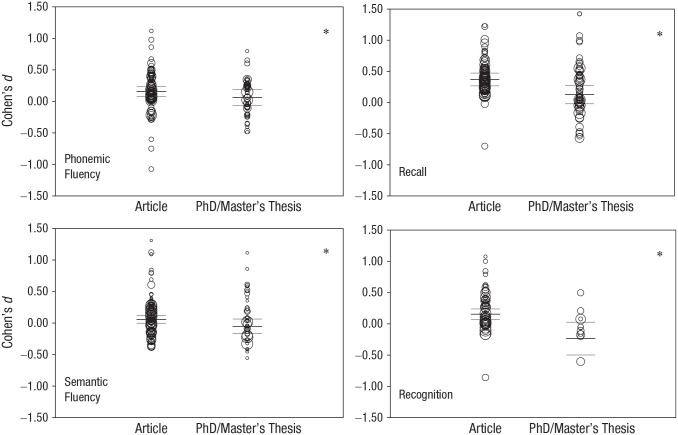
Effect of publication type. The asterisk denotes significant difference between published articles and PhD/master’s theses. Central lines represent means of the respective category, and upper and lower lines are confidence intervals. Figures are based on assuming perfect independence between multiple measures from the same sample or subsample.

Published articles consistently reported significantly higher female performance than PhD/master’s theses: phonemic fluency, *Z* = 2.00, *p* = .045, *B* = −0.093; semantic fluency, *Z* = 2.77, *p* = .006, *B* = −0.108; recall, *Z* = 4.01, *p* < .001, *B* = −0.243; and recognition, *Z* = 4.58, *p* < .001, *B* = −0.390 (see Fig. 2). Assuming perfect correlation, we found that all four effects remained significant.

#### Gender of first author

**Fig. 3.**
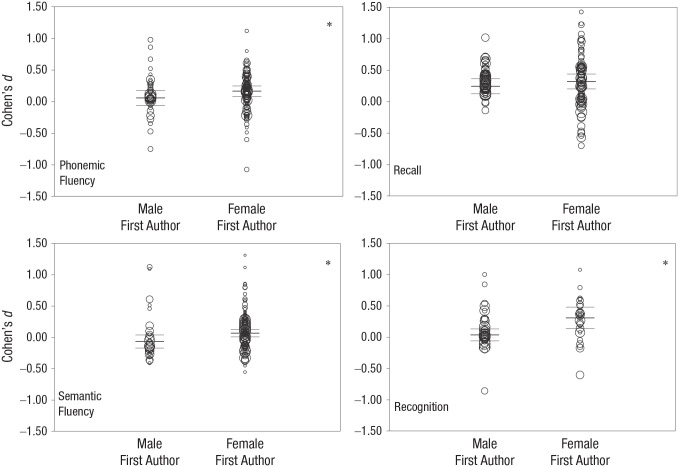
Gender of first-author effect. The asterisk denotes significant difference between female and male first authors. Central lines represent means of the respective category, and upper and lower lines are confidence intervals. Figures are based on assuming perfect independence between multiple measures from the same sample or subsample.

Female first authors reported significantly stronger female advantages in phonemic fluency (*Z* = 2.44, *p* = .015, *B* = 0.107), semantic fluency (*Z* = 3.69, *p* < .001, *B* = 0.134), and recognition (*Z* = 4.31, *p* < .001, *B* = 0.271) compared with male first authors (see Fig. 3). No significant difference between male and female first authors emerged in recall (*Z* = 1.36, *p* = .175, *B* = 0.076). Assuming perfect correlation, we found that all effects remained significant/nonsignificant.

#### Publication year

The female advantage significantly decreased in phonemic fluency (*Z* = 2.401, *p* = .016, *B* = −0.004) and recall (*Z* = 2.02, *p* = .044, *B* = −0.005) with publication year. However, the effect became nonsignificant in phonemic fluency if the oldest study (Elias, 1951) was removed (*Z* = 1.91, *p* = .057, *B* = −0.002). Neither semantic fluency (*Z* = 1.63, *p* = .103, *B* = −0.004) nor recognition (*Z* = 1.43, *p* = .152, *B* = −0.004) changed significantly with publication year (see Fig. S2 in the Supplemental Material). Assuming perfect correlation, we found that the effect in recall was no longer significant (*Z* = 1.73, *p* = .085, *B* = −0.005) and that all other effects remained nonsignificant (after removing Elias, 1951).

#### Mean age

In phonemic fluency, the female advantage became significantly smaller with increasing mean age (*Z* = 2.46, *p* = .014, *B* = −0.002). By contrast, the female advantage became significantly larger with increasing mean age in recall (*Z* = 2.07, *p* = .038, *B* = 0.002). However, the effect was nonsignificant (*Z* = 1.76, *p* = .078, *B* = 0.002) after removing the study with the oldest mean-age sample, which also had an unusually high female advantage (Bleecker et al., 1988). No significant mean-age effect emerged in semantic fluency (*Z* = 1.94, *p* = .052, *B* = −0.001) and recognition (*Z* = 0.05, *p* = .959, *B* < −0.001; see Fig. S3 in the Supplemental Material). Assuming perfect correlation, we found that the female advantage decreased significantly with age in semantic fluency (*Z* = 2.45, *p* = .014, *B* = −0.002) and increased significantly in recall also if Bleecker et al. (1988) was removed (*Z* = 2.03, *p* = .043, *B* = 0.002). All other effects remained significant/nonsignificant.

#### Age groups

A new set of metaregressions was computed that contained age groups and all significant covariates from the first set of metaregressions described above. Mean age was never retained because of multicollinearity with age groups.

The results are presented in Table 2. Age groups as a whole (i.e., with all age categories combined) varied significantly only in semantic fluency, *Q*(4) = 102.6, *p* < .001, based on 77 effect sizes. More specifically, the sex/gender difference in middle aged (*Z* = 2.01, *p* = .045, *B* = 0.093) and aged (*Z* = 7.65, *p* < .001, *B* = −0.273) differed significantly from the reference group, adults. There was no significant difference between child/child preschool or adolescent with adult (all *Z*s ≤ 1.57, all *p*s ≥ .117). Moreover, there were no significant overall effects of age groups in phonemic fluency, *Q*(4) = 5.49, *p* = .241, based on 63 effect sizes; recall, *Q*(4) = 7.54, *p* = .110, based on 67 effect sizes; and recognition, *Q*(4) = 6.85, *p* = .144, based on 35 effect sizes. In phonemic fluency (all *Z*s ≤ 1.56, all *p*s ≥ .119), also, none of the individual age groups differed significantly from the reference group, adult. In recall, the child/child preschool group had a significantly smaller female advantage than the adult group (*Z* = 2.15, *p* = .032, *B* = 0.200). In recognition, the adolescent (*Z* = 2.11, *p* = .035, *B* = 0.275) and child/child preschool (*Z* = 2.05, *p* = .040, *B* = 0.202) groups had a significantly higher female advantage than the adult reference group, but in the case of adolescents, this was based on only three effect sizes.

**Table 2.** Descriptive Overview of Age-Group Effects

	Phonemic fluency	**Semantic fluency**	Recall	Recognition
Child/child preschool (≤ 12 years)	*d* = 0.13 [0.06, 0.25], *k* = 29	*d* = 0.09 [−0.02, 0.17], *k* = 30	***d* = 0.05** [−0.06, 0.17], *k* = 15	***d* = 0.13** [−0.04, 0.31], *k* = 7
Adolescent (13–18 years)	*d* = 0.22 [0.03, 0.41], *k*=5	*d* = 0.03 [−0.25, 0.30], *k* = 2	*d* = 0.13 [−0.06, 0.31], *k* = 7	***d* = 0.11** [−0.14, 0.35], *k* = 3
Adult (19–44 years)	*d* = 0.24 [0.07, 0.41], *k* = 7	*d* = 0.15 [0.10, 0.21], *k* = 8	*d* = 0.28 [0.17, 0.39], *k* = 15	*d* = 0.02 [−0.10, 0.13], *k* = 9
Middle aged (45–64 years)	*d* = 0.13 [0.03, 0.23], *k* = 7	***d* = 0.25** [0.17, 0.32], *k* = 6	*d* = 0.34 [0.24, 0.45], *k* = 9	*d* = 0.13 [−0.04, 0.28], *k* = 6
Aged (≥ 65 years)	*d* = 0.06 [−0.03, 0.15], *k* = 15	*d* = −0.10* [−0.14, −0.07], *k* = 31	*d* = 0.17 [0.09, 0.24], *k* = 21	*d* = 0.06 [−0.09, 0.21], *k* = 10

Note: Values in parentheses represent 95% confidence intervals; *k* = number of effect sizes included. Boldface type indicates that individual age groups differed significantly from the reference group “adult.” Verbal-ability measures in boldface type indicate that the sex/gender difference varied significantly across all age groups. This table may contain more effect sizes than the metaregression because the metaregression includes only studies with information on all covariates. Values are based on assuming perfect independence between multiple measures from the same sample or subsample.

Assuming perfect correlation, we found that all age-groups effects in phonemic fluency (63 effect sizes) and semantic fluency (74 effect sizes) remained significant/nonsignificant. In recall, age groups as a whole remained nonsignificant, but now only the aged subsample had a significantly smaller female advantage than adult (*Z* = 2.30, *p* = .021, *B* = −0.127, based on 62 effect sizes). In recognition, age groups as a whole remained nonsignificant, and none of the individual age groups differed significantly from adults (all *Z*s ≤ 1.78, all *p*s ≥ .075, based on 26 effect sizes).

#### Gender of last author

A third set of metaregressions was computed for only published articles that contained last-author gender and all significant covariates from the respective first set of metaregressions. Publication type was not included because of multicollinearity. Last-author gender became significant only in semantic fluency (*Z* = 2.50, *p* < .001, *B* = −0.09, based on 90 effect sizes), in which male last authors reported a stronger female advantage than female last authors. No significant differences between male and female last authors emerged in phonemic fluency (*Z* = 1.68, *p* = .0093, *B* = 0.087, based on 72 effect sizes), recall (*Z* = 0.72, *p* = .474, *B* = 0.031, based on 70 effect sizes), and recognition (*Z* = 0.35, *p* = .729, *B* = −0.021, based on 53 effect sizes; see Fig. S4 in the Supplemental Material). Assuming perfect correlation, we found that all effects remained significant/nonsignificant.

## Discussion

Using a meta-analytical approach, we investigated whether women/girls perform better than men/boys in verbal fluency and verbal-episodic memory with neutral stimuli that were memorized intentionally and which factors moderated the female advantage.

### Small but robust female advantage in phonemic but not semantic fluency

Women/girls performed significantly better in phonemic fluency than men/boys (*d* = 0.13), but there was no significant female advantage in semantic fluency (*d*s = 0.01–0.02). When combined into a single verbal-fluency score, a significant female advantage remained (*d* = 0.07), but more by virtue of the large number of included effect sizes (*k* = 290). The female advantage is thus limited to phonemic fluency, and even here it is markedly lower than in the landmark meta-analysis by Hyde and Linn (1988), who reported a small effect (*d* = 0.33). This discrepancy might be partly due to a different definition of verbal fluency used in the present meta-analysis, which also included a much larger number of studies (168 vs. 14), thereby providing higher precision.

The overall effect size for phonemic fluency (*d*s = 0.12–0.13) is practically identical with both the COWAT/F-A-S (*d* = 0.14), the most frequently used test/starting-letter combination, and when generic starting letters or combination of generic starting letters are combined (*d* = 0.12). To illustrate the magnitude of the female advantage, if men/boys report a mean of 36 words, an effect of *d* = 0.14 would translate into an advantage of roughly 1.5 words for women/girls (*M* = 37.4) if a realistic standard deviation of 10 words is assumed.

The large number of studies and effect sizes in the present meta-analysis allowed testing whether the observed sex/gender difference in semantic fluency depended on the specific category participants were tasked with. The results revealed that men/boys generally named more animals (*d* = −0.13), whereas women/girls named more fruits/food/vegetables (*d* = 0.31). When both categories were combined, which several studies did, the effects size was slightly positive (*d* = 0.11), indicating a slight female advantage. These findings support the view that there is no overall female advantage in semantic fluency and that sex/gender differences are category-dependent (e.g., Laws, 2004; Sokołowski et al., 2020). Category dependency is also likely to account in part for the enormous heterogeneity in semantic fluency: The proportion of observed variance that reflects difference in true effect sizes (rather than sampling error) was 92%. Yet further research is needed to study those categories in more detail.

### Small but robust female advantage in verbal-episodic memory

We found a significant female advantage for verbal-episodic memory, in general, with effect sizes between *d* = 0.23 and *d* = 0.26. Furthermore, the female advantage was stronger in recall (*d* = 0.28) than in recognition (*d*s = 0.12–0.17). Both findings are in line with Asperholm et al. (2019), who reported an overall female advantage of *g* = 0.28 for episodic memory with verbal content and a female advantage for recall (*g*s = 0.28–0.31) and recognition (*g* = 0.17). Note that the studies included in both meta-analyses had only little overlap, which highlights the robustness of the female advantage. Recognition is generally considered easier than recall (e.g., Postman et al., 1948). Therefore, the female advantage might be smaller in the less difficult recognition tasks.

The strongest female advantage arose for the CVLT (*d* = 0.42) and the RAVLT (*d* = 0.39). By contrast, when the two tasks—delayed memory for names and visual-auditory learning—from the Woodcock Johnson-Psycho-Educational Battery–Revised were combined, there was a male advantage (*d* = −0.13). However, because all 12 effect sizes were taken from the same study (Cotten, 1991), generalization of these findings is questionable. In recognition, the CVLT (*d* = 0.17) and RAVLT (*d* = 0.22) also demonstrated a female advantage. The only task that showed a male advantage (i.e., storytelling delayed recognition; *d* = −0.07) was not significant (confidence bands include zero), and again all seven effect sizes were from the same study (Murre et al., 2013). To illustrate the magnitude of the female advantage in verbal-episodic memory, imagine a hypothetical study with the CVLT in which participants need to memorize a list with 16 nouns. If one assumes a realistic standard deviation of three words and *M* = 10 for men, Cohen’s *d* = 0.42 (the largest effect size found for verbal-episodic memory) translates into a female advantage of roughly one single word (*M* = 11.26).

Whereas the present meta-analysis together with Asperholm et al. (2019) suggest a small but robust female advantage for verbal-episodic memory, Voyer et al. (2021) demonstrated that the female advantage in verbal working memory is practically zero. The largest female advantage reported by the authors was *g* = 0.15 for free recall. This may be because certain tasks, which showed a reliable female advantage in the present study, for example the CVLT, were also included in Voyer et al. The distinction between episodic long-term and working memory is not always clear cut, and there are good arguments why the CVLT taps into both memory processes. In general, however, the findings from all three meta-analyses suggest that the female advantage in verbal memory is not universal and emerges especially when information needs to be transferred to long-term memory, whereas it is very small or absent in working memory.

### The female advantage is small but relevant

By comparison, the female advantage in verbal-episodic memory and phonemic fluency is smaller than in other verbal abilities, such as reading achievement (*d*s = 0.23–0.68; Reilly, 2012; Stoet & Geary, 2013) or writing abilities (*d*s = 0.53–0.61; Hedges & Nowell, 1995). In general, medium to large sex/gender differences were the exception, which is in line with the “gender-similarity hypothesis” (Hyde, 2005, 2014), according to which most sex/gender differences are in the small to medium range.

Verbal-episodic-memory and phonemic-fluency tasks are frequently used for assessing psychological impairments (Barker-Collo & Feigin, 2006; Collie & Maruff, 2000; Pennington & Ozonoff, 1996). Given that the present study corroborates previous findings that standard tests, such as CVLT (Kramer et al., 2003), RAVLT (Bleecker et al., 1988), and COWAT (Halari et al., 2005), reliably showed a female advantage, this implies that sex/gender should be taken into account when phonemic fluency and verbal-episodic memory are used in the clinical/diagnostic context.

### Stronger female advantage in published articles than PhD/master’s theses

We found support for the notion that the female advantage in verbal fluency and verbal-episodic memory is subject to publication bias. First, Egger’s regression and the funnel plots (see Fig. S1 in the Supplemental Material) suggest a “small study effect” for verbal-episodic memory, in general, as well as recall and recognition. That is, especially small studies with significant results favoring women/girls were more likely to be included in our meta-analysis than small studies favoring men/boys. Egger’s regression, however, was not significant for verbal, phonemic, or semantic fluency, which suggests the small-study effect is generally stronger in verbal-episodic memory.

In addition, we found that the female advantage in all four reported verbal abilities was higher in published articles than in PhD/master’s theses. The difference ranged between *d* = 0.09 and *d* = 0.39. In fact, for recognition, the female advantage was not significant in PhD/master’s theses. By using metaregressions, factors such as publication year, age, or first/last-author gender were controlled for. Therefore, it is unlikely that the publication-type effect was a mere artifact of, for instance, an overrepresentation of unpublished studies in a particular age group. Likewise, the publication bias is unlikely to arise from lower quality in non-peer-reviewed PhD/master’s theses: If this were the case, we would expect randomly weaker or larger sex/gender differences. However, we found consistently stronger female advantage in published articles. The most parsimonious explanation is therefore that studies are more likely to be published when they find the anticipated female advantage.

### First-authors’ gender affects sex/gender difference

The metaregression further revealed that the first-author’s gender affects the magnitude of the sex/gender difference in phonemic fluency, semantic fluency, and recognition, but not recall. Both male and female first authors consistently reported stronger performance for members of their own gender. The effect was in the range of *d*s = 0.11 to 0.27 and controlled for age, publication type, or publication year. Hyde and Linn (1988) reported a similar first-author bias but with smaller effect size (*d* = 0.07) and across a wide range of verbal abilities. We speculate that the first-author bias represents an in-group bias in which members of one’s own group are favored over out-group members. With these data, it is not possible to disentangle whether female first authors overreport or male first authors underreport the female advantage.

We also found a last-author effect in semantic fluency in which male last authors reported a significantly stronger female advantage than female last authors. This result is difficult to interpret because the sex/gender effect in semantic fluency is category-dependent, as described above. None of the other three measures (i.e., phonemic fluency, recall, and recognition) yielded significant last-author effects, and thus we refrain from speculations regarding last-author effects in the present study.

### No clear cohort or age effects

The female advantage decreased significantly with publication year for recall (when perfect independence between multiple outcomes was assumed), but the effect was small (*B* = −0.004) and did not emerge when perfect correlation was assumed. No significant effect was found for recognition (see also Asperholm et al., 2019). Likewise, the significant publication-year effect in phonemic fluency disappeared when one outlier was removed. Overall, sex/gender effects reported here were relatively stable over time.

Age effects were neither in line with the previously reported stronger deterioration in older men compared with older women (Graves et al., 2017; Kramer et al., 2003; Rodriguez-Aranda & Martinussen, 2006) nor with an inverted U-shaped curve with smaller sex/gender differences in earlier and later life (Asperholm et al., 2019). When the analysis was based on mean age, a significant coefficient (*B* = −0.002) was found only in phonemic fluency, which implies that the female advantage was reduced by *d* = 0.02 over a 10-year period—a small effect. When the analysis was based on age groups, none of the three verbal-ability measures that showed a reliable female advantage yielded a significant overall age-groups effect. In some cases, certain age groups differed significantly from the adult reference group (see Table 2), but most comparisons with adults were not significant. In general, findings for the three measures that yielded a female advantage indicated relatively stable sex/gender differences throughout life span (see also de Frias et al., 2006).

Semantic fluency was the only verbal domain that showed a significant overall age-group effect: Middle-aged participants (45–64, *d* = 0.25) showed the strongest female advantage, followed by adults (19–44, *d* = 0.15) and children (2–12, *d* = 0.09). Participants age 65 or older even showed a significant male advantage (*d* = −0.10). However, we refrain from interpretations because the female advantage was strongly category-dependent.

### Limitations

First, the statistical indicators showed considerable variance. The null hypothesis, according to which there is only one true underlying effect size, was violated in all analyses. To include data from very heterogeneous samples can be considered an asset because it increases the generalizability of our findings. However, although we investigated several moderator variables, there are other potentially relevant factors that we did not examine, such as (a) specific categories for semantic fluency, (b) test language, (c) monolingual versus bilingual participants, and (d) participants’ country/region of origin. The fact that most studies were carried out in the United States and United Kingdom and used native English-speaking participants might hamper generalizability. For example, a recent study did not find that the female advantage in phonemic fluency varied across countries, but only UK, Italy, and Norway were investigated (Moè et al., 2021). However, the female advantage in reading comprehension has been demonstrated to vary across countries (Reilly, 2012; Stoet & Geary, 2013).

Second, we analyzed age effects with two approaches (age means and age groups) that each have their advantages and disadvantages. Age means allowed including more effect sizes at the expense of precision because the single number of age mean becomes meaningless in samples with large age ranges. Age groups allowed examining sex/gender differences in clearly defined developmental periods but at the expense of losing effect sizes that do not fall in an age category. As a result, some of the age groups have very few effect sizes (e.g., two or three), and we thus refrained from interpreting too much into significant differences between specific age groups. Conducting those analyses seemed nevertheless justified, and the lack of clear age effects may in part be due to the complex nature of sex/gender differences across age.

Third, we contacted authors whose work we had already identified as suitable for our meta-analysis and where only key statistical parameters were missing for calculating effect sizes. We did not reach out to authors who simply used tests/tasks that we considered as adequate, and we also did not contact forums or researchers in the field of verbal fluency/memory. We further reached out only to authors who provided contact details in published articles, which were unavailable for authors of PhD/master’s theses. Moreover, we did not include data from Google Scholar because the massive numbers of reference (> 200,000) was simply unfeasible to process. Thus, although the present meta-analysis compiled a large body of data, we might have missed several primary studies.

### Conclusion and future avenues

Analyzing data from 168 studies, 496 effect sizes, and 355,173 participants, the present meta-analysis suggests that a small but robust female advantage in verbal fluency and verbal-episodic memory exists. With respect to verbal fluency, the female advantage emerged only in phonemic fluency, whereas sex/gender differences in semantic fluency appeared strongly category-dependent. The female advantage, especially in phonemic fluency, is smaller than previously shown (Hyde & Linn, 1988). However, phonemic fluency and verbal-episodic memory measures are frequently used in psychological/diagnostic settings, which highlights the need for taking sex/gender effects into account. A discussion of how the female advantage arises and what the underlying brain mechanisms are is beyond the scope of the present meta-analysis, but as argued for other cognitive sex/gender differences, we propose that the female advantage emerges from an intricate interaction of biological, psychological, and sociocultural factors (Halpern, 2012; Halpern & Tan, 2001; Hausmann, 2017; Jäncke, 2018).

The female advantage is affected by publication bias in two forms: Published articles reported larger female advantages than unpublished research, and both male and female first authors reported better performance for participants of their own gender. Although we found evidence for the existence of publication bias, it did not fully account for the female advantage reported here.

In general, meta-analyses focusing on cognitive abilities favoring women/girls are rare (for notable exceptions, see Asperholm et al., 2019; Voyer et al., 2007, 2021; Voyer & Voyer, 2014). Apart from including additional factors listed above, future studies should investigate publication bias and first-author/last-author effects in cognitive abilities in which men/boys typically excel (e.g., mental rotation). This has been largely ignored so far. Finally, more studies should adopt a biopsychosocial approach and include more routinely sex/gender-related, nonbinary factors (e.g., sex hormones, self-efficacy, gender stereotypes), and their interactions that might explain individual differences in verbal abilities and other cognitive domains better than sex/gender.

## Supplemental Material

sj-docx-1-pps-10.1177_17456916221082116 – Supplemental material for Sex/Gender Differences in Verbal Fluency and Verbal-Episodic Memory: A Meta-Analysis Click here for additional data file. Supplemental material, sj-docx-1-pps-10.1177_17456916221082116 for Sex/Gender Differences in Verbal Fluency and Verbal-Episodic Memory: A Meta-Analysis by Marco Hirnstein, Josephine Stuebs, Angelica Moè and Markus Hausmann in Perspectives on Psychological Science

## Transparency

*Action Editor:* Laura A. King

*Editor:* Laura A. King

## Notes

1. Cognitive differences between men/boys and women/girls arise from a complex interplay of biological, psychological, and sociocultural factors. These factors would be so intertwined that it would not be logical to distinguish between biology (“sex”) and social environment (“gender”). In the current study, we therefore aimed for a neutral terminology and avoided “sex” or “gender” as separate terms and instead used “sex/gender” whenever possible. In certain contexts, however, it would be inappropriate to use “sex/gender” when addressing specific biological or social constructs, such as gender equality, gender stereotypes, sex hormones, or sex chromosomes. When addressing first/last-author effects, we refer to gender because we identified authors as males or females simply on the basis of their first name, not knowing their biological sex or gender identity.
