# Supplementary material for: Sex/Gender Differences in Verbal Fluency and Verbal-Episodic Memory: A Meta-Analysis
Source: Perspect Psychol Sci. 2022 Jul 22;18(1):67–90. doi: 10.1177/17456916221082116 (PMC9896545; doi:10.1177/17456916221082116)
Supplement: sj-docx-1-pps-10.1177_17456916221082116 – Supplemental material for Sex/Gender Differences in Verbal Fluency and Verbal-Episodic Memory: A Meta-Analysis [file sj-docx-1-pps-10.1177_17456916221082116.docx]

# Supplementary material “Sex/Gender Differences in Verbal Fluency and Verbal Episodic Memory. A Meta-analysis”

# Table S1

*Terms used in Literature Search and Number of Identified References per Database*

| **Search terms** | **PubMed** | **ISI** | **PsycInfo** | **ProQuest** | **Total** |
| --- | --- | --- | --- | --- | --- |
| Gender + verbal fluency | 306 | 437 | 498 | 7931 | 9172 |
| Sex + verbal fluency | 435 | 481 | 640 | 8327 | 9883 |
| Gender + verbal memory | 288 | 385 | 355 | 8470 | 9498 |
| Sex + verbal memory | 475 | 537 | 421 | 7750 | 9183 |
| Gender + verbal ability | 79 | 132 | 306 | - | 517 |
| Sex + verbal ability | 149 | 166 | 17 | - | 332 |
| Female + Male + verbal fluency | 2808 | 80 | 198 | 9463 | 12549 |
| Female + male + verbal memory | 3399 | 97 | 163 | 9462 | 13121 |
| Female + Male + verbal ability | 592 | 26 | 327 | - | 945 |
| Women + Men + verbal fluency | 173 | 212 | 218 | - | 603 |
| Women + Men + verbal memory | 179 | 235 | 186 | - | 600 |
| Women + Men + verbal ability | 41 | 48 | 119 | - | 208 |
| Total | 8924 | 2836 | 3448 | 51403 | 66611 |

# Exclusion Criteria and Procedure during Abstract/Title Screening

During screening of the abstracts and titles, references were excluded if they (1) did not report behavioral data on verbal fluency/verbal episodic memory, (2) did not report empirical data (e.g., reviews, theoretical papers), (3) did not include data from men/boys *and* women/girls (i.e., no direct comparison of male/female performance), (4) reported data on animals or children too young to perform verbal fluency/memory tasks (e.g., 6 month olds), (5) had fewer than 10 male and 10 female participants (to mitigate the effect of spurious findings with very small sample sizes), (6) contained only clinical samples or samples with specific gene combinations; clinical studies with control groups could be retained unless controls were pre-selected such that they would match the clinical groups on factors that could affect verbal performance (e.g., intelligence, age, socioeconomic status), and (7) reported data only from experimental conditions (e.g., verbal fluency under emotional stress/stereotype threat); studies with control conditions could be retained.

The screening was performed by five raters. At first, all raters were given a training set of 100 randomly selected references. Based on the criteria above, each reference was classified as either included/excluded by each rater independently. Disagreements were then discussed among the raters and, when appropriate, classifications were changed. After a second classification round, all five raters agreed fully on 91% of the references, 4/5 raters agreed on 4% of references, and 3/5 raters agreed on 5% references. After training, all raters proceeded to screen their portion of the references alone.

# Table S2

*List of Studies/Effect Sizes Included in the Meta-analysis and their Key Characteristics*

| **Study** | **Total N (M/F)** | **Mean Age (Range)** | **Type of verbal ability** | **Verbal task** | **Language** | **1st author gender** | **Last author gender** | **Cohen's d** |
| --- | --- | --- | --- | --- | --- | --- | --- | --- |
| [Abdel Aziz et al. (2017)](#_ENREF_1)^a^ | 139 (76/63) | 51.3 (20-93) | Phonemic | Starting letters: T, R, W, Q, D, S | Arabic | M | M | -0.15 |
|  | 139 (76/63) | 51.3 (20-93) | Semantic | Animals, Objects | Arabic | M | M | -0.29 |
| [Acevedo et al. (2000)](#_ENREF_2)^a^ | 37 (7/30) | 54.5 (50-59) | Semantic | Animals, Fruits/vegetables/food | English | F | M | 1.31 |
|  | 64 (15/49) | 54.5 (50-59) | Semantic | Animals, Fruits/vegetables/food | Spanish | F | M | 0.69 |
|  | 107 (30/77) | 64.5 (60-69) | Semantic | Animals, Fruits/vegetables/food | English | F | M | 0.79 |
|  | 97 (32/65) | 64.5 (60-69) | Semantic | Animals, Fruits/vegetables/food | Spanish | F | M | 0.29 |
|  | 172 (45/127) | 74.5 (70-79) | Semantic | Animals, Fruits/vegetables/food | English | F | M | 0.25 |
|  | 76 (26/50) | 74.5 (70-79) | Semantic | Animals, Fruits/vegetables/food | Spanish | F | M | 0.32 |
| [Agard (2008)](#_ENREF_3)^p^ | 95 (47/48) | 43.06 (18-70) | Recall | CVLT | English | F | - | 0.34 |
| [Alexiou (2005)](#_ENREF_5)^p^ | 73 (41/32) | 6.04 (5-7) | Recall | 10 Word Learning Test | Greek | F | - | 0.08 |
| [Ardila et al. (2005)](#_ENREF_7)^a^ | 622 (276/346) | 9.5 (5-14) | Semantic | Animals | Spanish | M | F | -0.16 |
| [Auriacombe et al. (2010)](#_ENREF_9)^a^ | 1132 (459/673) | 77.8 (65-100) | Phonemic | Starting letters: P, L | English | F | M | -0.22 |
|  | 1132 (459/673) | 77.8 (65-100) | Semantic | Animals, Fruits/vegetables/food, Cities | English | F | M | 0.01 |
| [Banks et al. (1987)](#_ENREF_12)^a^ | 60 (23/37) | 69.2 (65-75) | Recall | Selective Reminding Test | English | F | M | 0.89 |
| [Baxter (1998)](#_ENREF_15)^p^ | 104 (52/52) | 23.5 (18-45) | Recall | RAVLT | English | F | - | 0.52 |
| [Blair (2002)](#_ENREF_19)^p^ | 25 (15/10) | 32.3 (18-60) | Recall | CVLT | English | F | - | 1.42 |
| [Bleecker et al. (1988)](#_ENREF_20)^a^ | 31 (15/16) | (40-49) | Recall | RAVLT | English | F | F | 0.12 |
|  | 31 (15/16) | (40-49) | Recognition | RAVLT | English | F | F | 0.60 |
|  | 42 (20/22) | (50-59) | Recall | RAVLT | English | F | F | 0.57 |
|  | 42 (20/22) | (50-59) | Recognition | RAVLT | English | F | F | 0.41 |
|  | 52 (23/29) | (60-69) | Recall | RAVLT | English | F | F | 0.82 |
|  | 52 (23/29) | (60-69) | Recognition | RAVLT | English | F | F | 0.62 |
|  | 47 (18/29) | (70-79) | Recall | RAVLT | English | F | F | 0.79 |
|  | 47 (18/29) | (70-79) | Recognition | RAVLT | English | F | F | 0.79 |
|  | 24 (11/13) | (80-89) | Recall | RAVLT | English | F | F | 1.24 |
|  | 24 (11/13) | (80-89) | Recognition | RAVLT | English | F | F | 1.08 |
| [Bolla et al. (1990)](#_ENREF_23)^a^ | 199 (80/119) | 64.3 (39-89) | Phonemic | COWAT/F, A, S | English | F | F | 0.35 |
| [Bolla et al. (1998)](#_ENREF_22)^a^ | 196 (135/61) | 64.9 (55-69) | Phonemic | COWAT/F, A, S | English | F | F | 0.06 |
|  | 196 (135/61) | 64.9 (55-69) | Semantic | Animals, Fruits/vegetables/food | English | F | F | 0.33 |
|  | 195 (125/70) | 74.5 (70-79) | Phonemic | COWAT/F, A, S | English | F | F | 0.14 |
|  | 195 (125/70) | 74.5 (70-79) | Semantic | Animals, Fruits/vegetables/food | English | F | F | 0.32 |
|  | 87 (45/42) | 83.5 (80-94) | Phonemic | COWAT/F, A, S | English | F | F | -0.17 |
|  | 87 (45/42) | 83.5 (80-94) | Semantic | Animals, Fruits/vegetables/food | English | F | F | 0.19 |
| [Bolla‐Wilson and Bleecker (1986)](#_ENREF_21)^a^ | 114 (55/59) | 62.0 (40-84) | Recall | RAVLT | English | F | F | 0.55 |
|  | 114 (55/59) | 62.0 (40-84) | Recognition | RAVLT | English | F | F | 0.36 |
| [Brandling-Bennett (2006)](#_ENREF_27)^p^ | 111 (56/55) | 9.5 (6-13) | Recall | Generic word list | English | F | - | 0.06 |
|  | 111 (56/55) | 9.5 (6-13) | Semantic | Animals, Fruits/vegetables/food | English | F | - | -0.12 |
| [Brocki and Bohlin (2004)](#_ENREF_28)^a^ | 23 (14/9) | 12.1 (11.6-13) | Semantic | Animals, Fruits/vegetables/food | Swedish | F | F | 0.40 |
|  | 25 (11/14) | 7.0 (6-7.5) | Semantic | Animals, Fruits/vegetables/food | Swedish | F | F | 0.31 |
|  | 23 (12/11) | 9.0 (7.5-9.5) | Semantic | Animals, Fruits/vegetables/food | Swedish | F | F | 0.39 |
|  | 21 (10/11) | 10.5 (9.6-11.5) | Semantic | Animals, Fruits/vegetables/food | Swedish | F | F | 0.17 |
| [Brosnan (1973)](#_ENREF_29)^p^ | 72 (36/36) | 5.5 (4-7) | Recall | Generic word list | English | M | - | -0.01 |
|  | 72 (36/36) | 5.5 (4-7) | Recognition | Generic word list | English | M | - | -0.21 |
| [Brucki and Rocha (2004)](#_ENREF_31)^a^ | 257 (105/152) | 49.4 (16-88) | Semantic | Animals | Portuguese | F | F | -0.12 |
| [Burton and Henninger (2013)](#_ENREF_33)^a^ | 182 (56/126) | 19.6 (adults) | Phonemic | Starting letters: S, C (Thurstone Word Fluency Test) | English | F | F | 0.44 |
| [Capitani et al. (1998)](#_ENREF_34)^a^ | 102 (43/59) | 23.5 (18-29) | Phonemic | Starting letters: P, W, F | Italian | M | F | 0.03 |
|  | 83 (41/42) | 34.5 (30-39) | Phonemic | Starting letters: P, W, F | Italian | M | F | 0.05 |
|  | 99 (50/49) | 44.5 (40-49) | Phonemic | Starting letters: P, W, F | Italian | M | F | 0.05 |
|  | 95 (39/56) | 54.5 (50-59) | Phonemic | Starting letters: P, W, F | Italian | M | F | 0.30 |
|  | 97 (35/62) | 64.5 (60-69) | Phonemic | Starting letters: P, W, F | Italian | M | F | 0.27 |
|  | 27 (13/14) | 75.5 (70-81) | Phonemic | Starting letters: P, W, F | Italian | M | F | 0.43 |
| [Carstairs et al. (2012)](#_ENREF_35)^a^ | 390 (188/202) | 25.6 (18-34) | Recognition | RAVLT | English | F | M | 0.37 |
|  | 390 (188/202) | 25.6 (18-34) | Recall | RAVLT | English | F | M | 0.53 |
| [Cerhan et al. (1998)](#_ENREF_37)^a^ | 1521 (624/897) | 47.0 (45-49) | Phonemic | COWAT/F, A, S | English | M | M | 0.08 |
|  | 1521 (624/897) | 47.0 (45-49) | Recall | Delayed Word Recall (10 words) | English | M | M | 0.40 |
|  | 3783 (1598/2185) | 52.0 (50-54) | Phonemic | COWAT/F, A, S | English | M | M | 0.11 |
|  | 3783 (1598/2185) | 52.0 (50-54) | Recall | Delayed Word Recall (10 words) | English | M | M | 0.36 |
|  | 3552 (1542/2010) | 57.0 (55-59) | Phonemic | COWAT/F, A, S | English | M | M | 0.08 |
|  | 3552 (1542/2010) | 57.0 (55-59) | Recall | Delayed Word Recall (10 words) | English | M | M | 0.36 |
|  | 3232 (1508/1724) | 62.0 (60-64) | Phonemic | COWAT/F, A, S | English | M | M | 0.06 |
|  | 3232 (1508/1724) | 62.0 (60-64) | Recall | Delayed Word Recall (10 words) | English | M | M | 0.33 |
|  | 1709 (846/863) | 67.0 (65-69) | Phonemic | COWAT/F, A, S | English | M | M | 0.02 |
|  | 1709 (846/863) | 67.0 (65-69) | Recall | Delayed Word Recall (10 words) | English | M | M | 0.25 |
| [Chan and Poon (1999)](#_ENREF_38)^a^ | 316 (133/183) | (7-95) | Semantic | Animals | Chinese | F | F | -0.35 |
|  | 316 (133/183) | (7-95) | Semantic | Vehicles/transportation | Chinese | F | F | -0.38 |
| [Chan et al. (2003)](#_ENREF_39)^a^ | 36 (16/20) | (16-26) | Semantic | Animals, Food, Means of Transport, Furniture | Chinese | Blank | F | 0.36 |
|  | 55 (26/29) | (27-65) | Semantic | Animals, Food, Means of Transport, Furniture | Chinese | Blank | F | -0.15 |
| [Chang (1992)](#_ENREF_40)^m^ | 20 (10/10) | 23.7 (21-27) | Recall | 48 high and low-imagery words | English | F | - | 0.05 |
| [Chipman (1998)](#_ENREF_42)^m^ | 48 (24/24) | 21.0 (19-28) | Recall | CVLT | English | F | - | 0.64 |
|  | 49 (25/24) | 23.3 (19-33) | Recall | CVLT | English | F | - | 0.97 |
|  | 48 (24/24) | 22.0 (19-36) | Recall | CVLT | English | F | - | 0.58 |
| [Cohen (1975)](#_ENREF_43)^p^ | 33 (13/20) | 62.0 (60-64) | Phonemic | Word Fluency (Christensen & Guildford) - words containing certain letters | English | F | - | -0.05 |
|  | 65 (37/28) | 67.0 (65-69) | Phonemic | Word Fluency (Christensen & Guildford) - words containing certain letters | English | F | - | 0.29 |
|  | 62 (31/31) | 72.0 (70-74) | Phonemic | Word Fluency (Christensen & Guildford) - words containing certain letters | English | F | - | 0.35 |
| [Cole (1991)](#_ENREF_44)^p^ | 190 (62/128) | 20.0 (undergrads) | Phonemic | COWAT/F, A, S | English | M | - | 0.06 |
| [Comilang (2003)](#_ENREF_46)^p^ | 124 (32/92) | 19.9 (18-26) | Phonemic | COWAT/C, F, L | English | F | - | 0.26 |
| [Contador et al. (2016)](#_ENREF_47)^a^ | 1908 (843/1065) | 72.1 (67-74) | Semantic | Animals, Fruits/vegetables/foods | Spanish | M | M | -0.05 |
|  | 2078 (888/1190) | 75.3 (75-79) | Semantic | Animals, Fruits/vegetables/foods | Spanish | M | M | -0.14 |
|  | 1248 (511/737) | 79.8 (80-84) | Semantic | Animals, Fruits/vegetables/foods | Spanish | M | M | -0.14 |
|  | 633 (257/376) | 84.1 (85+) | Semantic | Animals, Fruits/vegetables/foods | Spanish | M | M | -0.22 |
| [Corona-LoMonaco (2000)](#_ENREF_48)^p^ | 78 (43/35) | 40.5 (16-65) | Phonemic | COWAT/F, A, S | Spanish | F | - | -0.08 |
|  | 78 (43/35) | 40.5 (16-65) | Recall | RAVLT | Spanish | F | - | 0.04 |
|  | 78 (43/35) | 40.5 (16-65) | Recognition | RAVLT | Spanish | F | - | -0.15 |
|  | 78 (43/35) | 40.5 (16-65) | Semantic | Animals | Spanish | F | - | -0.27 |
|  | 78 (43/35) | 40.5 (16-65) | Semantic | Fruits/vegetables/food | Spanish | F | - | -0.10 |
| [Cory (2003)](#_ENREF_49)^p^ | 80 (27/53) | 39.3 (20-74) | Phonemic | COWAT (PMR/CFL) | English & Spanish | M | - | -0.47 |
|  | 80 (27/53) | 39.3 (20-74) | Recall | CVLT | English & Spanish | M | - | -0.14 |
| [Cotten (1991)](#_ENREF_50)^p^ | 53 (28/25) | (Grade 10) | Recall | Delayed Memory for Names, Visual-Auditory Learning from Woodcock Johnson Psycho-Educational Battery Revised | English | F | - | 0.15 |
|  | 144 (69/75) | (Grade 11) | Recall | Delayed Memory for Names, see above | English | F | - | -0.25 |
|  | 66 (28/38) | (Grade 12) | Recall | Delayed Memory for Names, see above | English | F | - | 0.09 |
|  | 100 (52/48) | (Grade 2) | Recall | Delayed Memory for Names, see above | English | F | - | -0.39 |
|  | 218 (109/109) | (Grade 3) | Recall | Delayed Memory for Names, see above | English | F | - | -0.07 |
|  | 74 (40/34) | (Grade 4) | Recall | Delayed Memory for Names, see above | English | F | - | 0.12 |
|  | 151 (78/73) | (Grade 5) | Recall | Delayed Memory for Names, see above | English | F | - | -0.24 |
|  | 89 (42/47) | (Grade 6) | Recall | Delayed Memory for Names, see above | English | F | - | -0.50 |
|  | 81 (42/39) | (Grade 7) | Recall | Delayed Memory for Names, see above | English | F | - | -0.48 |
|  | 163 (73/90) | (Grade 8) | Recall | Delayed Memory for Names, see above | English | F | - | 0.02 |
|  | 44 (21/23) | (Grade 9) | Recall | Delayed Memory for Names, see above | English | F | - | 0.03 |
| [Crossley et al. (1997)](#_ENREF_51)^a^ | 628 (258/370) | (65-85) | Phonemic | COWAT/F, A, S | English | F | M | 0.26 |
|  | 635 (258/377) | (65-85) | Semantic | Animals | English | F | M | -0.15 |
| [Dadin et al. (2009)](#_ENREF_53)^a^ | 39 (20/19) | 18.9 (Under-  grads) | Recall | RAVLT | Spanish | F | F | 0.52 |
| [de Frias et al. (2006)](#_ENREF_54)^a^ | 625 (286/339) | 52.8 (35-80) | Recall | Generic word list | Swedish | F | F | 0.38 |
|  | 625 (286/339) | 52.8 (35-80) | Recognition | Generic word list | Swedish | F | F | 0.27 |
| [DeWan (2006)](#_ENREF_57)^p^ | 75 (39/36) | 8.0 (8) | Verbal fluency | Verbal Fluency Test from D-KEFS | English | F | - | 0.07 |
|  | 75 (36/39) | 9.0 (9) | Verbal fluency | Verbal Fluency Test from D-KEFS | English | F | - | 0.36 |
|  | 75 (40/35) | 10.0 (10) | Verbal fluency | Verbal Fluency Test from D-KEFS | English | F | - | 0.30 |
|  | 75 (38/37) | 11.0 (11) | Verbal fluency | Verbal Fluency Test from D-KEFS | English | F | - | 0.39 |
|  | 100 (47/53) | 12.0 (12) | Verbal fluency | Verbal Fluency Test from D-KEFS | English | F | - | 0.56 |
|  | 100 (50/50) | 13.0 (13) | Verbal fluency | Verbal Fluency Test from D-KEFS | English | F | - | 0.48 |
|  | 100 (50/50) | 14.0 (14) | Verbal fluency | Verbal Fluency Test from D-KEFS | English | F | - | 0.20 |
|  | 100 (46/54) | 15.0 (15) | Verbal fluency | Verbal Fluency Test from D-KEFS | English | F | - | 0.13 |
| [Dias et al. (2013)](#_ENREF_58)^a^ | 35 (15/20) | 6.0 (6) | Phonemic | COWAT/F, A, S | Portuguese | F | F | 1.12 |
|  | 87 (36/51) | 7.0 (7) | Phonemic | COWAT/F, A, S | Portuguese | F | F | 0.41 |
|  | 95 (50/45) | 8.0 (8) | Phonemic | COWAT/F, A, S | Portuguese | F | F | 0.49 |
|  | 63 (36/27) | 9.0 (9) | Phonemic | COWAT/F, A, S | Portuguese | F | F | -0.29 |
|  | 76 (50/26) | 10.0 (10) | Phonemic | COWAT/F, A, S | Portuguese | F | F | 0.13 |
|  | 47 (21/26) | 11.0 (11) | Phonemic | COWAT/F, A, S | Portuguese | F | F | -0.24 |
|  | 42 (19/23) | 12.0 (12) | Phonemic | COWAT/F, A, S | Portuguese | F | F | 0.19 |
|  | 61 (35/26) | 13.0 (13) | Phonemic | COWAT/F, A, S | Portuguese | F | F | 0.43 |
|  | 66 (38/28) | 14.0 (14) | Phonemic | COWAT/F, A, S | Portuguese | F | F | 0.07 |
| [Egelko (1983)](#_ENREF_62)^p^ | 50 (25/25) | 65.0 (45-85) | Phonemic | Starting letters: F, A, S, J, U | English | F | - | 0.25 |
| [Elias (1951)](#_ENREF_64)^p^ | 288 (155/133) | 12.2 (11-14) | Phonemic | Starting letter: S | English | M | - | 0.35 |
| [Ernest (1983)](#_ENREF_65)^a^ | 209 (72/137) | 19.8 | Recognition | Concrete Word Recognition Memory task | English | F | - | 0.39 |
|  | 209 (72/137) | 19.8 | Semantic | Associations to four concrete and abstract words (Paivio,1971) | English | F | - | -0.15 |
| [Farace (1996)](#_ENREF_66)^p^ | 157 (80/77) | 20.2 (18-35) | Phonemic | COWAT (F, A, S) | English | F | - | 0.25 |
| [Fares (2011)](#_ENREF_67)^p^ | 78 (38/40) | 44.7 (20-80) | Phonemic | Starting letters: Hah, Meem, Teh, | Arabic | F | - | -0.21 |
|  | 78 (38/40) | 44.7 (20-80) | Phonemic | COWAT/F, A, S | English | F | - | -0.10 |
|  | 78 (38/40) | 44.7 (20-80) | Semantic | Animals | Arabic | F | - | -0.18 |
|  | 78 (38/40) | 44.7 (20-80) | Semantic | Animals | English | F | - | -0.17 |
| [Faul (2008)](#_ENREF_68)^p^ | 17875 (7771/10104) | 64.1 | Recall | 10 Word Learning Test from CERAD | English | F | - | 0.04 |
| [Findlay et al. (2010)](#_ENREF_70)^a^ | 11067 (4989/6078) | 60.0 (51-69) | Recall | RAVLT | English & French | F | F | 0.24 |
|  | 12478 (5678/6800) | 60.0 (51-69) | Semantic | Animals | English & French | F | F | -0.03 |
|  | 6362 (2383/3979) | (70+) | Recall | RAVLT | English & French | F | F | 0.19 |
|  | 9061 (3518/5543) | (70+) | Semantic | Animals | English & French | F | F | -0.11 |
| [Fraser (1986)](#_ENREF_71)^p^ | 108 (55/53) | 12.0 (11-13) | Recall | List of nonsense and English words | English | M | - | 0.50 |
| [Gates (1986)](#_ENREF_74)^p^ | 24 (12/12) | 10.6 | Phonemic | Starting letter: B | English | F | - | -0.41 |
|  | 24 (12/12) | 10.6 | Semantic | Food | English | F | - | -0.42 |
|  | 24 (12/12) | 6.6 | Phonemic | Starting letter: B | English | F | - | 0.80 |
|  | 24 (12/12) | 6.6 | Semantic | Food | English | F | - | 0.04 |
| [Gavin (1988)](#_ENREF_75)^p^ | 200 (100/100) | 57.0 (17-97) | Phonemic | Starting letters: S, C | English | F | - | 0.21 |
| [Gawda and Szepietowska (2013a)](#_ENREF_76)^a^ | 302 (164/138) | 31.7 (18-70) | Semantic | Animals | Polish | F | F | 0.10 |
|  | 302 (164/138) | 31.7 (18-70) | Semantic | Vehicles/transportation | Polish | F | F | 0.03 |
| [Gawda and Szepietowska (2013b)](#_ENREF_77)^a^ | 200 (71/129) | 37.5 (18-70) | Semantic | Animals | Polish | F | F | -0.29 |
|  | 200 (71/129) | 37.5 (18-70) | Semantic | Fruit | Polish | F | F | 0.16 |
| [Gerdeman (1975)](#_ENREF_78)^p^ | 74 (39/35) | (Undergrads) | Recognition | Recognition of quotes from a filmed interview | English | M | - | -0.08 |
| [González et al. (2005)](#_ENREF_79)^a^ | 593 (263/329) | 60.0 (60+) | Semantic | Animals | English | M | F | -0.22 |
|  | 684 (280/404) | 60.0 (60+) | Semantic | Animals | Spanish | M | F | -0.27 |
| [Gould (1972)](#_ENREF_80)^p^ | 212 (37/175) | 35.0 (20-50) | Phonemic | Four Word Sentences | English | F | - | -0.02 |
| [Greenstein et al. (2010)](#_ENREF_82)^a^ | 812 (416/396) | 13.5 (10-17) | Recall | RAVLT | Hebrew | M | F | 0.23 |
|  | 812 (416/396) | 13.5 (10-17) | Recognition | RAVLT | Hebrew | M | F | 0.05 |
| [Gregory (2002)](#_ENREF_83)^m^ | 64 (32/32) | 20.9 (18-30) | Recall | List of professions with 32 words | English | F | - | -0.02 |
| [Greshner (2000)](#_ENREF_84)^m^ | 47 (24/23) | 20.5 (Undergrads) | Recall | CVLT | English | F | - | 0.26 |
| [Gur et al. (2001)](#_ENREF_86)^a^ | 92 (48/44) | 29.0 (18-69) | Recall | CVLT | English | M | F | 0.45 |
|  | 92 (48/44) | 29.0 (18-69) | Recall | Logical Memory II from Wechsler Memory Scale | English | M | F | 0.71 |
| [Halari (2003)](#_ENREF_88)^p^ | 84 (42/42) | 28.0 (19-35) | Phonemic | COWAT/F, A, S | English | F | - | 0.23 |
|  | 84 (42/42) | 28.0 (19-35) | Semantic | Animals, Fruits/vegetables/foods | English | F | - | 0.61 |
| [Harrison et al. (2000)](#_ENREF_95)^a^ | 365 (199/166) | 40.8 | Phonemic | Starting letter: B | English | M | M | 0.02 |
|  | 365 (199/166) | 40.8 | Semantic | Animals | English | M | M | 0.10 |
| [Hausmann et al. (2009)](#_ENREF_99)^a^ | 56 (31/25) | 24.7 | Phonemic | Four Word Sentences | German | M | F | 0.86 |
|  | 56 (31/25) | 24.7 | Phonemic | Starting letters: P, L | German | M | F | 0.67 |
| [Havlena (1990)](#_ENREF_100)^m^ | 32 (16/16) | (4th and 7^th^ graders) | Recall | 24 atypical category items | English | F | - | 0.47 |
| [Hazin et al. (2016)](#_ENREF_101) | 304 (134/170) | 7.15 (7-10) | Phonemic | Starting letters: F, A, M | Portuguese | F | F | -0.16 |
|  |  |  | Semantic | Animals, fruits/vegetables/food, clothing | Portuguese | F | F | 0.27 |
| [Heister (1982)](#_ENREF_103)^a^ | 64 (32/32) | 23.5 (19-35) | Phonemic | Starting letters: S, M | German | F | - | 0.57 |
|  | 64 (32/32) | 23.5 (19 to 35) | Semantic | Things “round” and “red” | German | F | - | 0.10 |
| [Held (2013)](#_ENREF_104)^p^ | 40 (20/20), Caucasian Americans | 42.8 (18-76) | Semantic | animals, boys' names from D-KEFS | English | M | - | -0.05 |
|  | 40 (20/20), Filipino Americans | 42.8 (18-76) | Semantic | animals, boys' names from D-KEFS | English | M | - | 0.51 |
|  | 40 (20/20), Caucasian Americans | 44.5 (18-83) | Phonemic | COWAT/F, A, S | English | M | - | -0.10 |
|  | 40 (20/20), Filipino Americans | 44.5 (18-83) | Phonemic | COWAT/F, A, S | English | M | - | 0.14 |
| [Hendrawan et al. (2015)](#_ENREF_105)^a^ | 211 (80/131) | (Undergrads) | Phonemic | Starting letters: 24 Indonesian letters | Indonesian | M | M | -0.13 |
| [Herlitz et al. (1997)](#_ENREF_107)^a^ | 1000 (470/530) | 57.5 (35-80) | Phonemic | Five letter words starting with M | Swedish | F | M | 0.17 |
|  | 1000 (470/530) | 57.5 (35-80) | Phonemic | Starting letter: A | Swedish | F | M | 0.15 |
|  | 1000 (470/530) | 57.5 (35-80) | Recall | Generic word list | Swedish | F | M | 0.25 |
| [Herlitz et al. (1999)](#_ENREF_106)^a^ | 200 (100/100) | 30.0 (20-40) | Phonemic | COWAT/F, A, S | Swedish | F | F | 0.50 |
|  | 200 (100/100) | 30.0 (20-40) | Recall | List of abstract and concrete words | Swedish | F | F | 0.24 |
|  | 200 (100/100) | 30.0 (20-40) | Recognition | List of abstract and concrete words | Swedish | F | F | 0.14 |
| [Herlitz et al. (2013)](#_ENREF_108)^a^ | 187 (102/85) | 13.0 (12-14) | Phonemic | COWAT/F, A, S | Swedish | F | F | 0.61 |
|  | 187 (102/85) | 13.0 (12-14) | Recall | Generic word list | Swedish | F | F | 0.31 |
| [Hirnstein et al. (2012)](#_ENREF_110)^a^ | 39 (21/18) | 32.5 (18-47) | Phonemic | Combined score for starting letters L, P and Four Word Sentences | German | M | M | 0.52 |
| [Hirnstein et al. (2014)](#_ENREF_109)^a^ | 70 (34/36) | 25.0 (19-50) | Phonemic | Four Word Sentences | German | M | M | -0.75 |
|  | 70 (34/36) | 25.0 (19-50) | Phonemic | Starting letters: L, P | German | M | M | 0.98 |
| [Holland (1969)](#_ENREF_113)^p^ | 112 (52/60) | (High school students) | Phonemic | Words beginning with one letter and ending with another | English | F | - | 0.18 |
| [Hurks (2013)](#_ENREF_114)^a^ | 80 (40/40) | 10.7 (8-12) | Semantic | Animals | Dutch | F | - | 0.22 |
| [Isomura (2002)](#_ENREF_118)^p^ | 60 (30/30) | 27.5 (18-35) | Recall | CVLT | English | F | - | 0.43 |
| [Iverson et al. (2014)](#_ENREF_119)^a^ | 100 (50/50) | 35.8 (18-68) | Recall | Generic word list | English | M | M | 0.35 |
| [John and Rajashekhar (2014)](#_ENREF_121)^a^ | 214 (107/107) | 14.1 (13-15) | Semantic | Animals | Malayalam | F | M | 0.08 |
|  | 202 (103/99) | 6.4 (5-8) | Semantic | Animals | Malayalam | F | M | 0.06 |
|  | 200 (101/99) | 8.6 (7-10) | Semantic | Animals | Malayalam | F | M | 0.03 |
|  | 201 (102/99) | 10.3 (9-11) | Semantic | Animals | Malayalam | F | M | -0.20 |
|  | 198 (99/99) | 12.2 (11-13) | Semantic | Animals | Malayalam | F | M | -0.01 |
| [John et al. (2016)](#_ENREF_122)^a^ | 198 (99/99) | 12.2 (11-13) | Phonemic | Starting letters: P, K | Malayalam | F | M | 0.04 |
|  | 214 (107/107) | 14.1 (13-15) | Phonemic | Starting letters: P, K | Malayalam | F | M | 0.10 |
|  | 202 (103/99) | 6.4 (5-8) | Phonemic | Starting letters: P, K | Malayalam | F | M | 0.44 |
|  | 200 (101/99) | 8.6 (7-10) | Phonemic | Starting letters: P, K | Malayalam | F | M | 0.25 |
|  | 201 (102/99) | 10.3 (9-11) | Phonemic | Starting letters: P, K | Malayalam | F | M | 0.23 |
| [Johnston (1965)](#_ENREF_123)^p^ | 142 (31/111) | (Undergrads) | Semantic | Things "round" | English | M | - | -0.02 |
|  | 142 (31/111) | (Undergrads) | Semantic | Things "blue" | English | M | - | 0.12 |
| [Jordan (2014)](#_ENREF_124)^m^ | 43 (23/20), Bilinguals | (55+) | Phonemic | Starting letter: P | English | F | - | 0.13 |
|  | 43 (23/20), Bilinguals | (55+) | Semantic | Animals | English | F | - | -0.47 |
|  | 53 (18/35), Monolinguals | (55+) | Phonemic | Starting letter: P | English | F | - | -0.02 |
|  | 53 (18/35), Monolinguals | (55+) | Semantic | Animals | English | F | - | 0.07 |
|  | 43 (23/20) |  | Phonemic | Starting letter: P | Lakota | F | - | 0.10 |
|  | 43 (23/20) |  | Semantic | Animals | Lakota | F | - | -0.74 |
| [Kavé (2005)](#_ENREF_127)^a^ | 269 (115/154) | 51.5 (18-85) | Phonemic | Starting letters: bet, gimeal, shin | Hebrew | F | - | 0.05 |
|  | 369 (155/214) | 51.5 (18-85) | Semantic | Animals, Fruits/vegetables/food, Transportation/Vehicles | Hebrew | F | - | -0.12 |
| [Kemmotsu (2010)](#_ENREF_128)^p^ | 71 (31/40) | 65.0 (45-89), Bilinguals | Phonemic | COWAT/F, A, S | English | F | - | 0.60 |
|  | 71 (31/40) | 65.0 (45-89), Bilinguals | Semantic | Animals | English | F | - | 0.59 |
|  | 71 (31/40) | 65.0 (45-89), Bilinguals | Recall total | CVLT | English | F | - | 1.07 |
|  | 71 (34/37) | 64.6 (45-91), Monolinguals | Phonemic | COWAT/F, A, S | English | F | - | 0.03 |
|  | 71 (34/37) | 64.6 (45-91), Monolinguals | Semantic | Animals | English | F | - | 0.24 |
|  | 71 (34/37) | 64.6 (45-91), Monolinguals | Recall total | CVLT | English | F | - | 0.25 |
| [Kempler et al. (1998)](#_ENREF_129)^a^ | 317 (112/205) | 76.5 (54-99) | Semantic | Animals | Chinese, Spanish, Vietnamese, English | M | F | -0.36 |
| [Kesse-Guyot et al. (2012)](#_ENREF_130)^a^ | 1079 (631/448) | (>70) | Phonemic | Starting letter: P | French | F | F | 0.11 |
|  | 1079 (631/448) | (>70) | Semantic | Animals | French | F | F | 0.03 |
|  | 863 (137/726) | 53.5 (48-59) | Phonemic | Starting letter: P | French | F | F | 0.40 |
|  | 863 (137/726) | 53.5 (48-59) | Semantic | Animals | French | F | F | 0.15 |
|  | 3104 (1588/1516) | 64.5 (60-69) | Phonemic | Starting letter: P | French | F | F | 0.18 |
|  | 3104 (1588/1516) | 64.5 (60-69) | Semantic | Animals | French | F | F | 0.02 |
| [Khalil (2010)](#_ENREF_131)^a^ | 215 (125/90) | (17-59) | Phonemic | Starting letters: W, R, G | Arabic | M | - | -0.22 |
| [Kim and Kang (1999)](#_ENREF_132)^a^ | 60 (30/30) | 25 (20-29) | Recall | CVLT | Korean | M | M | 0.40 |
| [Kimura and Clarke (2002)](#_ENREF_134)^a^ | 85 (41/44) |  | Recall | Generic word list | English | F | M | 0.55 |
| [Kimura and Seal (2003)](#_ENREF_135)^a^ | 53 (25/28) | 20 (Undergrads) | Recall | Lists with nonsense, concrete, and abstract words | English | F | F | -0.70 |
| [Knaus (2003)](#_ENREF_137)^p^ | 25 (13/12) | 26.9 (19-41) | Phonemic | COWAT/F, A, S | English | F | - | 0.27 |
|  | 25 (13/12) | 26.9 (19-41) | Semantic | Animals, Fruits/vegetables/food, First names - from Woodcock-Johnson III Tests of Cognitive Abilities | English | F | - | 0.58 |
| [Knight et al. (2006)](#_ENREF_138)^a^ | 272 (122/150) | 73.7 (65-90) | Recall | RAVLT | English | M | M | 0.43 |
|  | 272 (122/150) | 73.7 (65-90) | Recognition | RAVLT | English | M | M | 0.28 |
|  | 272 (122/150) | 73.7 (65-90) | Semantic | Animals | English | M | M | 0.04 |
|  | 272 (122/150) | 73.7 (65-90) | Semantic | Fruits/vegetables/food | English | M | M | 0.61 |
|  | 272 (122/150) | 73.7 (65-90) | Semantic | Vehicles/transportation | English | M | M | -0.12 |
| [Kosmidis et al. (2004)](#_ENREF_140)^a^ | 65 (31/34) | 24.9 (18-39) | Phonemic | Starting letters: chi, alpha, sigma (equivalent of F, A, S) | Greek | F | M | -0.09 |
|  | 65 (31/34) | 24.9 (18-39) | Semantic | Animals, Fruits, Objects | Greek | F | M | -0.01 |
|  | 42 (14/28) | 49.1 (40-59) | Phonemic | Starting letters: chi, alpha, sigma (equivalent of F, A, S) | Greek | F | M | 0.13 |
|  | 42 (14/28) | 49.1 (40-59) | Semantic | Animals, Fruits, Objects | Greek | F | M | 0.37 |
|  | 46 (16/30) | 65.5 (60-77) | Phonemic | Starting letters: chi, alpha, sigma (equivalent of F, A, S) | Greek | F | M | -0.28 |
|  | 46 (16/30) | 65.5 (60-77) | Semantic | Animals, Fruits, Objects | Greek | F | M | 0.45 |
| [Kramer et al. (1997)](#_ENREF_141)^a^ | 121 (72/49) | 12.0 (11-12) | Recall | CVLT | English | M | F | 0.22 |
|  | 121 (72/49) | 12.0 (11-12) | Recognition | CVLT | English | M | F | 0.05 |
|  | 108 (60/48) | 14.0 (13-14) | Recall | CVLT | English | M | F | 0.51 |
|  | 108 (60/48) | 14.0 (13-14) | Recognition | CVLT | English | M | F | 0.29 |
|  | 120 (56/64) | 16.1 (15-16) | Recall | CVLT | English | M | F | 0.39 |
|  | 120 (56/64) | 16.1 (15-16) | Recognition | CVLT | English | M | F | 0.00 |
|  | 132 (66/66) | 6.1 (5-6) | Recall | CVLT | English | M | F | 0.16 |
|  | 132 (66/66) | 6.1 (5-6) | Recognition | CVLT | English | M | F | 0.30 |
|  | 145 (71/74) | 8.0 (7-8) | Recall | CVLT | English | M | F | 0.21 |
|  | 145 (71/74) | 8.0 (7-8) | Recognition | CVLT | English | M | F | 0.12 |
|  | 147 (76/71) | 10.0 (9-10) | Recall | CVLT | English | M | F | 0.30 |
|  | 147 (76/71) | 10.0 (9-10) | Recognition | CVLT | English | M | F | 0.11 |
| [Kramer et al. (2003)](#_ENREF_142) | 446 (201/245) | 72.2 (55-89) | Recall | CVLT | English | M | M | 0.60 |
|  | 446 (201/245) | 72.2 (55-89) | Recognition | CVLT | English | M | M | 0.50 |
|  | 573 (288/285) | 28.3 (16-47) | Recall | CVLT | English | M | M | 0.43 |
|  | 573 (288/285) | 28.3 (16-47) | Recognition | CVLT | English | M | M | 0.42 |
| [LaFevor (2017)](#_ENREF_147)^p^ | 188 (147/41) | 9.4 (6-11) | Recall | Word Memory Subtest from ImPACT | English | F | - | -0.04 |
|  | 188 (147/41) | 9.4 (6-11) | Recognition | Word Memory Subtest from ImPACT | English | F | - | -0.11 |
| [Lanting et al. (2009)](#_ENREF_148)^a^ | 60 (29/31) | 29.0 (18-40) | Phonemic | COWAT/C, F, L | English | F | F | 0.00 |
|  | 60 (29/31) | 29.0 (18-40) | Semantic | Animals | English | F | F | 0.24 |
|  | 72 (29/43) | 78.0 (65 - 91) | Phonemic | COWAT/F, A, S | English | F | F | 0.35 |
|  | 72 (29/43) | 78.0 (65-91) | Semantic | Animals | English | F | F | -0.10 |
| [Larrabee and Crook (1993)](#_ENREF_149)^a^ | 384 (192/192) | 18.0 (17-19) | Recall | Grocery List Selective Reminding Task | English | M | M | 0.68 |
|  | 124 (62/62) | 24.5 (20-29) | Recall | Grocery List Selective Reminding Task | English | M | M | 0.66 |
|  | 118 (59/59) | 44.5 (30-59) | Recall | Grocery List Selective Reminding Task | English | M | M | 1.01 |
|  | 148 (74/74) | 64.5 (60-69) | Recall | Grocery List Selective Reminding Task | English | M | M | 0.42 |
|  | 60 (30/30) | (70+) | Recall | Grocery List Selective Reminding Task | English | M | M | 1.02 |
| [Laws (2004)](#_ENREF_150)^a^ | 600 (300/300) | 30.8 (Adults) | Semantic | Animals, Vehicles/Transportation, Clothing, Fruits/vegetables/food | English | M | - | 0.00 |
| [Lero (1974)](#_ENREF_151)^p^ | 130 (64/66) | 11.1 (5th  graders) | Phonemic | Instances (Wallach & Kogan, 1965) | English | F | - | 0.31 |
| [Lewin et al. (2001)](#_ENREF_154)^a^ | 185 (91/94) | 29.4 (20-40) | Phonemic | Starting letters: F, A, S, N | Swedish | F | F | 0.16 |
| [Liang (2013)](#_ENREF_155)^p^ | 100 (58/42) | 20.4 (16-30) | Recall | Logical Memory II from Wechsler Memory Scale | Chinese | F | - | 0.54 |
|  | 100 (58/42) | 20.4 (16-30) | Recall | Logical Memory II from Wechsler Memory Scale | Chinese | F | - | 0.38 |
|  | 100 (58/42) | 20.4 (16-30) | Recognition | Logical Memory Recognition from Wechsler Memory Scale | Chinese | F | - | 0.50 |
|  | 100 (58/42) | 20.4 (16-30) | Recognition | Word list from Wechsler Memory Scale III | Chinese | F | - | 0.21 |
|  | 100 (58/42) | 20.4 (16-30) | Semantic | Fruits/vegetables/food | Chinese | F | - | 0.47 |
| [Lindblad (1995)](#_ENREF_156)^p^ | 701 (352/349) | 3.5 (2-5) | Recall | Delayed Memory for Names/Visual-Auditory Learning from Woodcock Johnson Psycho-Educational Battery - Revised | English | F | - | 0.02 |
| [Lowe (1998)](#_ENREF_158)^m^ | 38 (19/19) | 10.1 (8-12) | Phonemic | Starting letters: S, C (Thurstone Word Fluency Test) | English | F | - | 0.23 |
| [Lundervold et al. (2014)](#_ENREF_160)^a^ | 158 (52/106) | 61.3 | Recall total | CVLT | Norwegian | F | F | 0.96 |
|  | 158 (52/106) | 61.3 | Recognition | CVLT | Norwegian | F | F | 0.22 |
| [Mathuranath et al. (2003)](#_ENREF_164)^a^ | 153 (62/91) | 66.9 (55-84) | Phonemic | Starting letters: P, A | Malayalam | M | M | -0.28 |
|  | 153 (62/91) | 66.9 (55-84) | Semantic | Animals | Malayalam | M | M | -0.21 |
| [Maylor et al. (2007)](#_ENREF_165)^a^ | 50688 (26640/24048) | 22.0 (20-24) | Semantic | Objects Colored Grey | unclear but mostly English | F | M | 0.10 |
|  | 44162 (23941/20221) | 26.8 (25-29) | Semantic | Objects Colored Grey | unclear but mostly English | F | M | 0.12 |
|  | 32243 (18188/14055) | 31.9 (30-34) | Semantic | Objects Colored Grey | unclear but mostly English | F | M | 0.15 |
|  | 23328 (13470/9858) | 36.8 (35-39) | Semantic | Objects Colored Grey | unclear but mostly English | F | M | 0.16 |
|  | 17227 (9852/7375) | 41.8 (40-44) | Semantic | Objects Colored Grey | unclear but mostly English | F | M | 0.20 |
|  | 12628 (7109/5519) | 46.9 (45-49) | Semantic | Objects Colored Grey | unclear but mostly English | F | M | 0.21 |
|  | 9287 (5232/4055) | 51.7 (50-54) | Semantic | Objects Colored Grey | unclear but mostly English | F | M | 0.24 |
|  | 5647 (3373/2274) | 56.7 (55-59) | Semantic | Objects Colored Grey | unclear but mostly English | F | M | 0.27 |
|  | 2911 (1807/1104) | 61.9 (60-65) | Semantic | Objects Colored Grey | unclear but mostly English | F | M | 0.29 |
| [McKay (1995)](#_ENREF_166)^p^ | 108 (61/47) | 27.0 (7-47) | Semantic | Animals, Fruits/vegetables/food, Parts of a House, Parts of Nature, Vehicles/Transportation, Clothing | English | F | - | 0.52 |
| [Mohd (1997)](#_ENREF_172)^p^ | 150 (75/75) | 7.0 (7) | Semantic | Fruits/vegetables/food | Arabic | F | - | 0.19 |
| [Moulden (1992)](#_ENREF_175)^m^ | 38 (19/19) | 10.6 (10-11) | Phonemic | Starting letters: P, C, S | English | M | - | 0.25 |
|  | 38 (19/19) | 10.6 (10-11) | Semantic | Animals, Objects | English | M | - | -0.41 |
|  | 36 (17/19) | 12.5 (12-13) | Phonemic | Starting letters: P, C, S | English | M | - | 0.28 |
|  | 36 (17/19) | 12.5 (12-13) | Semantic | Animals, Objects | English | M | - | -0.26 |
|  | 30 (14/16) | 14.5 (14-15) | Phonemic | Starting letters: P, C, S | English | M | - | 0.34 |
|  | 30 (14/16) | 14.5 (14-15) | Semantic | Animals, Objects | English | M | - | -0.32 |
|  | 53 (22/31) | 6.8 (6-7) | Phonemic | Starting letters: P, C, S | English | M | - | 0.20 |
|  | 53 (22/31) | 6.8 (6-7) | Semantic | Animals, Objects | English | M | - | -0.05 |
|  | 43 (19/24) | 8.5 (8-9) | Phonemic | Starting letters: P, C, S | English | M | - | -0.35 |
|  | 43 (19/24) | 8.5 (8-9) | Semantic | Animals, Objects | English | M | - | 0.04 |
| [Mullins (1977)](#_ENREF_176)^m^ | 144 (61/83) | 20.0 (17-23) | Phonemic | Starting letter: M | English | F | - | 0.25 |
| [Munnelly (2016)](#_ENREF_177)^m^ | 100 (50/50) | (Undergrads) | Recall | Generic word list | English | F | - | 0.14 |
| [Murre et al. (2013)](#_ENREF_178)^a^ | 1232 (314/918) | 16.1 (11-18) | Recall | 10 Word Learning Test | Dutch | M | M | 0.34 |
|  | 1467 (368/1099) | 16.1 (11-18) | Recall | Deese Roediger McDermott task | Dutch | M | M | 0.11 |
|  | 977 (237/740) | 16.1 (11-18) | Recognition | Deese Roediger McDermott immediate recall | Dutch | M | M | 0.09 |
|  | 973 (231/742) | 16.1 (11-18) | Recognition | Story telling delayed recognition | Dutch | M | M | 0.01 |
|  | 1520 (390/1130) | 16.1 (11-18) | Recognition | Story telling immediate recognition | Dutch | M | M | 0.00 |
|  | 3026 (977/2049) | 23.9 (19-30) | Recall | 10 Word Learning Test | Dutch | M | M | 0.23 |
|  | 3439 (1093/2346) | 23.9 (19-30) | Recall | Deese Roediger McDermott task | Dutch | M | M | 0.12 |
|  | 2407 (720/1687) | 23.9 (19-30) | Recognition | Deese Roediger McDermott immediate recall | Dutch | M | M | 0.03 |
|  | 2368 (735/1633) | 23.9 (19-30) | Recognition | Story telling delayed recognition | Dutch | M | M | 0.01 |
|  | 3136 (998/2138) | 23.9 (19-30) | Recognition | Story telling immediate recognition | Dutch | M | M | 0.01 |
|  | 1688 (594/1094) | 35.5 (31-40) | Recall | 10 Word Learning Test | Dutch | M | M | 0.15 |
|  | 1904 (659/1245) | 35.5 (31-40) | Recall | Deese Roediger McDermott task | Dutch | M | M | 0.15 |
|  | 1360 (447/913) | 35.5 (31-40) | Recognition | Deese Roediger McDermott immediate recall | Dutch | M | M | 0.16 |
|  | 1447 (520/927) | 35.5 (31-40) | Recognition | Story telling delayed recognition | Dutch | M | M | -0.06 |
|  | 1787 (631/1156) | 35.5 (31-40) | Recognition | Story telling immediate recognition | Dutch | M | M | 0.04 |
|  | 2141 (668/1453) | 45.7 (41-50) | Recall | 10 Word Learning Test | Dutch | M | M | 0.27 |
|  | 2289 (722/1567) | 45.7 (41-50) | Recall | Deese Roediger McDermott task | Dutch | M | M | 0.10 |
|  | 1561 (464/1097) | 45.7 (41-50) | Recognition | Deese Roediger McDermott immediate recall | Dutch | M | M | 0.17 |
|  | 1926 (632/1294) | 45.7 (41-50) | Recognition | Story telling delayed recognition | Dutch | M | M | -0.16 |
|  | 2332 (764/1568) | 45.7 (41-50) | Recognition | Story telling immediate recognition | Dutch | M | M | 0.04 |
|  | 1843 (614/1229) | 54.9 (51-60) | Recall | 10 Word Learning Test | Dutch | M | M | 0.30 |
|  | 2113 (666/1447) | 54.9 (51-60) | Recall | Deese Roediger McDermott task | Dutch | M | M | 0.11 |
|  | 1309 (390/919) | 54.9 (51-60) | Recognition | Deese Roediger McDermott immediate recall | Dutch | M | M | 0.11 |
|  | 1826 (618/1208) | 54.9 (51-60) | Recognition | Story telling delayed recognition | Dutch | M | M | -0.11 |
|  | 2191 (738/1453) | 54.9 (51-60) | Recognition | Story telling immediate recognition | Dutch | M | M | 0.03 |
|  | 606 (236/370) | 64.4 (61-70) | Recall | 10 Word Learning Test | Dutch | M | M | 0.22 |
|  | 739 (284/455) | 64.4 (61-70) | Recall | Deese Roediger McDermott task | Dutch | M | M | 0.21 |
|  | 420 (152/268) | 64.4 (61-70) | Recognition | Deese Roediger McDermott immediate recall | Dutch | M | M | 0.26 |
|  | 598 (241/357) | 64.4 (61-70) | Recognition | Story telling delayed recognition | Dutch | M | M | -0.17 |
|  | 699 (277/422) | 64.4 (61-70) | Recognition | Story telling immediate recognition | Dutch | M | M | 0.01 |
|  | 214 (107/107) | 74.6 (71-80) | Recall | 10 Word Learning Test | Dutch | M | M | 0.33 |
|  | 250 (113/137) | 74.6 (71-80) | Recall | Deese Roediger McDermott task | Dutch | M | M | 0.32 |
|  | 108 (44/64) | 74.6 (71-80) | Recognition | Deese Roediger McDermott immediate recall | Dutch | M | M | 0.54 |
|  | 197 (101/96) | 74.6 (71-80) | Recognition | Story telling delayed recognition | Dutch | M | M | 0.02 |
|  | 257 (142/115) | 74.6 (71-80) | Recognition | Story telling immediate recognition | Dutch | M | M | 0.01 |
| [Nida (1986)](#_ENREF_180)^p^ | 72 (36/36) | 4.5 (4-5) | Recall | List of 12 line drawings | English | M | - | 0.45 |
| [O'Hara et al. (2006)](#_ENREF_181)^a^ | 163 (62/101) | 71.1 (60-100) | Recall | Logical Memory II from Wechsler Memory Scale | English | F | M | 0.23 |
| [Pedersen (2005)](#_ENREF_183)^p^ | 93 (54/39) | 3.5 (3-4) | Semantic | Animals, Fruits/vegetables/foods | English | F | - | 0.20 |
| [Phillips (1977)](#_ENREF_185)^m^ | 60 (30/30) | 6.2 (5-6) | Semantic | Animals, things to eat, wear, and ride | English | F | - | 0.08 |
| [Pino Escobar (2017)](#_ENREF_186)^m^ | 34 (14/20) | 7.8 (7-8) | Phonemic | Starting letter: F | English | F | - | 0.29 |
|  | 34 (14/20) | 7.8 (7-8) | Semantic | Animals | English | F | - | 0.10 |
| [Prigatano et al. (2008)](#_ENREF_188)^a^ | 213 (83/130) | 10.0 (6 to 14) | Semantic | Animals | English | M | F | -0.38 |
| [Rae (1979)](#_ENREF_190)^p^ | 89 (41/48) | 10.3 (9-11) | Recall | Generic word list | English | M | - | 0.65 |
| [Rahman et al. (2003)](#_ENREF_191)^a^ | 120 (60/60) | 28.4 (18-40) | Phonemic | Starting letters: P, R, W | English | M | M | 0.17 |
|  | 120 (60/60) | 28.4 (18-40) | Semantic | Animals, Fruits/vegetables/food | English | M | M | 1.12 |
| [Ratcliff et al. (2003)](#_ENREF_192)^a^ | 348 (138/210) | (65-74) | Phonemic | Starting letters: B, S | English | M | F | 0.04 |
|  | 348 (138/210) | (65-74) | Recall | 10 Word Learning Test | English | M | F | 0.23 |
|  | 348 (138/210) | (65-74) | Semantic | Animals, Fruits/vegetables/food | English | M | F | 0.18 |
|  | 77 (25/52) | (75+) | Phonemic | Starting letters: B, S | English | M | F | -0.01 |
|  | 77 (25/52) | (75+) | Recall | 10 Word Learning Test | English | M | F | 0.65 |
|  | 77 (25/52) | (75+) | Semantic | Animals, Fruits/vegetables/food | English | M | F | -0.04 |
| [Renteria (2005)](#_ENREF_194)^p^ | 53 (30/23) | 29.2 (25-34) | Phonemic | COWAT (PMR/CFL) | Spanish | F | - | -0.36 |
|  | 47 (20/27) | 43.0 (35-54) | Phonemic | Starting letters: P, M, R, C, F, L | Spanish | F | - | -0.21 |
| [Reynolds (1967)](#_ENREF_195)^m^ | 85 (54/31) | (Undergrads) | Phonemic | Four Word Sentences | English | M | - | 0.16 |
|  | 85 (54/31) | (Undergrads) | Phonemic | Starting letter: S | English | M | - | -0.04 |
|  | 85 (54/31) | (Undergrads) | Semantic | Things Category Test | English | M | - | -0.14 |
| [Riva et al. (2000)](#_ENREF_196)^a^ | 153 (78/75) | 8.5 (6-11) | Phonemic | Starting letters: B, S | Italian | F | F | 0.13 |
|  | 153 (78/75) | 8.5 (6-11) | Semantic | Animals, Fruits/vegetables/food | Italian | F | F | 0.07 |
| [Rosen (1995)](#_ENREF_198)^p^ | 33 (19/14) | 6.9 (5-8) | Semantic | Animals, Vehicles/Transporation, Clothing, Fruits/vegetables/food | English | F | - | -0.06 |
| [Rosselli, Ardila, et al. (2009)](#_ENREF_199)^a^ | 248 (109/139) | 14.8 (13-16) | Phonemic | Starting letter: M | Spanish | F | F | 0.29 |
|  | 278 (122/156) | 9.1 (7-10) | Phonemic | Starting letter: M | Spanish | F | F | 0.10 |
| [Rosselli, Tappen, et al. (2009)](#_ENREF_200)^a^ | 105 (29/76) | 76.5 (55 - 98) | Semantic | Animals, Fruits/vegetables/food, Clothing | Spanish | F | F | 0.23 |
| [Rouch et al. (2005)](#_ENREF_201)^a^ | 896 (431/465) | 32.0 (32) | Verbal Memory | RAVLT | French | F | M | 0.48 |
|  | 972 (488/484) | 42.0 (42) | Verbal Memory | RAVLT | French | F | M | 0.53 |
|  | 899 (461/438) | 52.0 (52) | Verbal Memory | RAVLT | French | F | M | 0.30 |
|  | 470 (280/190) | 62.0 (62) | Verbal Memory | RAVLT | French | F | M | 0.38 |
| [Rubin (2009)](#_ENREF_202)^p^ | 57 (27/30) | 27.7 (18-40) | Recall | CVLT | English | F |  | 0.72 |
| [Ryan et al. (2004)](#_ENREF_203)^a^ | 262 (157/105) | 19.8 (17-27) | Phonemic | COWAT/F, A, S | English | F | F | 0.13 |
| [Sakamoto (2009)](#_ENREF_204)^p^ | 30 (15/15) | 75.0 (65-85) | Phonemic | Starting letters: shi, i, re (equivalent of F, A, S) | Japanese | F | - | 0.18 |
|  | 30 (15/15) | 75.0 (65-85) | Recall total | PVLT | Japanese | F | - | -0.05 |
|  | 30 (15/15) | 75.0 (65-85) | Recognition | PVLT | Japanese | F | - | 0.08 |
|  | 30 (15/15) | 75.0 (65-85) | Semantic | Animals | Japanese | F | - | -0.56 |
| [Sakurai et al. (2011)](#_ENREF_205)^a^ | 41 (16/25) | 74.6 | Semantic | Animals | Japanese | M | M | -0.22 |
|  | 41 (16/25) | 74.6 | Semantic | Fruits/vegetables/food | Japanese | M | M | 1.09 |
| [Sandel (2016)](#_ENREF_206)^p^ | 499 (310/189) | 15.4 (13-18) | Recognition | Word Memory Subtest from ImPACT | English | F | - | 0.08 |
| [Schallmo et al. (2015)](#_ENREF_207)^a^ | 40 (21/19) | 38.4 (18-65) | Recall | CVLT | English | M | M | 0.18 |
|  | 40 (21/19) | 38.4 (18-65) | Recognition | CVLT | English | M | M | 1.00 |
| [Sims (2007)](#_ENREF_211)^p^ | 106 (48/58) | 44.3 (21-73) | Recall | CVLT | English | F | - | 0.43 |
| [Sinay (1967)](#_ENREF_212)^p^ | 156 (82/74) |  | Phonemic | Word Fluency (Christensen & Guildford) - words containing certain letters | English | F | - | 0.14 |
| [Snitz et al. (2009)](#_ENREF_213)^a^ | 1117 (398/719) | 72.0 (65-79) | Semantic | Animals | English | F | F | -0.03 |
|  | 740 (251/489) | 87.5 (80-95) | Semantic | Animals | English | F | F | -0.25 |
| [Soleman et al. (2013)](#_ENREF_215)^a^ | 51 (25/26) | 14.6 (11-17) | Phonemic | Starting letters: K, O, M, D, A, T | Dutch | M | F | 0.16 |
|  | 51 (25/26) | 14.6 (11-17) | Semantic | Animals, Fruits/vegetables/food, Clothing | Dutch | M | F | 0.46 |
| [Sosa et al. (2009)](#_ENREF_216)^a^ | 2022 (890/1132), China | (65>) | Recall | 10 Word Learning Test from CERAD battery | Chinese | F | M | -0.12 |
|  | 2022 (890/1132), China | (65>) | Semantic | Animals | Chinese | F | M | -0.24 |
|  | 2621 (933/1688), Cuba | (65>) | Recall | 10 Word Learning Test from CERAD battery | Spanish | F | M | 0.05 |
|  | 2621 (933/1688), Cuba | (65>) | Semantic | Animals | Spanish | F | M | -0.31 |
|  | 1769 (613/1156), Dominican Republic | (65>) | Recall | 10 Word Learning Test from CERAD battery | Spanish | F | M | 0.21 |
|  | 1769 (613/1156), Dominican Republic | (65>) | Semantic | Animals | Spanish | F | M | -0.23 |
|  | 1821 (822/999), India | (65>) | Recall | 10 Word Learning Test from CERAD battery | India (not further specified) | F | M | -0.10 |
|  | 1821 (822/999), India | (65>) | Semantic | Animals | India (not further specified) | F | M | -0.41 |
|  | 1823 (679/1144), Mexico | (65>) | Recall | 10 Word Learning Test from CERAD battery | Spanish | F | M | 0.26 |
|  | 1823 (679/1144), Mexico | (65>) | Semantic | Animals | Spanish | F | M | -0.12 |
|  | 1767 (694/1073), Peru | (65>) | Recall | 10 Word Learning Test from CERAD battery | Spanish | F | M | 0.29 |
|  | 1767 (694/1073), Peru | (65>) | Semantic | Animals | Spanish | F | M | -0.08 |
|  | 1826 (666/1160), Venezuela | (65>) | Recall | 10 Word Learning Test from CERAD battery | Spanish | F | M | 0.20 |
|  | 1826 (666/1160), Venezuela | (65>) | Semantic | Animals | Spanish | F | M | -0.09 |
| [Speer et al. (2014)](#_ENREF_217)^a^ | 150 (53/97) | 55.6 (50-60) | Recall | RAVLT | German | F | M | 0.58 |
|  | 150 (53/97) | 55.6 (50-60) | Recognition | RAVLT | German | F | M | 0.52 |
|  | 257 (122/135) | 65.3 (61-70) | Recall | RAVLT | German | F | M | 0.46 |
|  | 257 (122/135) | 65.3 (61-70) | Recognition | RAVLT | German | F | M | 0.39 |
| [Stanulis (1977)](#_ENREF_219)^p^ | 30 (16/14) | 19.2 (18-23) | Recall | List of abstract and concrete words | English | M | - | 0.11 |
| [Stoddard (2007)](#_ENREF_221)^p^ | 58 (32/26) | 19.0 (18-26) | Recall | Logical Memory II from Wechsler Memory Scale | English | F | - | 0.09 |
|  | 58 (32/26) | 19.0 (18-26) | Recall | Word list from Wechsler Memory Scale III | English | F | - | 0.39 |
| [Sunderaraman et al. (2013)](#_ENREF_224)^a^ | 62 (26/36) | 66.1 | Phonemic | COWAT/C, F, L | English | F | F | -1.07 |
|  | 48 (21/27) | 67.9 | Phonemic | COWAT/F, A, S | English | F | F | 0.15 |
|  | 62 (26/36) | 66.1 | Recall | CVLT | English | F | F | 1.22 |
|  | 48 (21/27) | 67.9 | Recall | PVLT | English | F | F | 0.70 |
| [Sundermann et al. (2016)](#_ENREF_225)^a^ | 379 (192/187) | 74.2 (55-90) | Recall | RAVLT | English | F | F | 0.32 |
| [Swift (1999)](#_ENREF_226)^p^ | 72 (24/48) | 38.0 (18-75) | Recall | CVLT | Spanish | M | - | 0.18 |
|  | 72 (24/48) | 38.0 (18-75) | Recognition | CVLT | Spanish | M | - | -0.05 |
| [Tallberg et al. (2008)](#_ENREF_227)^a^ | 49 (24/25) | 22.5 (16-29) | Phonemic | COWAT/F, A, S | Swedish | F | M | -0.60 |
|  | 49 (24/25) | 22.5 (16-29) | Semantic | Action Verbs | Swedish | F | M | -0.24 |
|  | 49 (24/25) | 22.5 (16-29) | Semantic | Animals | Swedish | F | M | 0.07 |
|  | 61 (34/27) | 47.0 (30-64) | Phonemic | COWAT/F, A, S | Swedish | F | M | 0.13 |
|  | 61 (34/27) | 47.0 (30-64) | Semantic | Action Verbs | Swedish | F | M | 0.29 |
|  | 61 (34/27) | 47.0 (30-64) | Semantic | Animals | Swedish | F | M | 0.26 |
|  | 55 (25/30) | 77.0 (65-89) | Phonemic | COWAT/F, A, S | Swedish | F | M | 0.37 |
|  | 55 (25/30) | 77.0 (65-89) | Semantic | Action Verbs | Swedish | F | M | 0.84 |
|  | 55 (25/30) | 77.0 (65-89) | Semantic | Animals | Swedish | F | M | 0.14 |
| [Tanaka (2005)](#_ENREF_228)^p^ | 72 (36/36) | 67.5 (50-85) | Recall | CVLT | English | F | - | 1.00 |
|  | 72 (36/36) | 67.5 (50-85) | Recognition | CVLT | English | F | - | -0.05 |
| [Temple and Cornish (1993)](#_ENREF_229)^a^ | 128 (64/64) | 10.0 (9-11) | Recognition | Warrinton Recognition Memory Battery | English | F | F | 0.59 |
| [Thilers et al. (2007)](#_ENREF_230)^a^ | 347 (182/165) | 60.0 (35-90) | Phonemic | Starting letter: A, 5 letter words starting with M | Swedish | F | F | 0.23 |
| [Thomas et al. (1978)](#_ENREF_231)^a^ | 72 (36/36) | 23.2 (5-60) | Semantic | Colours | Nepali | F | M | 0.80 |
| [Thornburg (1973)](#_ENREF_232)^p^ | 70(30/40) | 26.2 (2nd year college students) | Phonemic | Word Endings | English | M | - | 0.23 |
|  | 70 (30/40) | 26.2 (2nd year college students) | Phonemic | Four Word Sentences | English | M | - | 0.08 |
| [Tombaugh et al. (1999)](#_ENREF_234)^a^ | 1300 (559/741) | 50.5 (16-85) | Phonemic | COWAT/F, A, S | English | M | F | 0.06 |
|  | 735 (310/425) | 55.5 (16-95) | Semantic | Animals | English | M | F | -0.18 |
| [Tuck (2012)](#_ENREF_236)^p^ | 23 (10/13) | 5.0 (5) | Phonemic | COWAT/F, A, S | English | F | - | -0.03 |
|  | 23 (10/13) | 5.0 (5) | Semantic | Animals, Fruits/vegetables/food, Action Verbs | English | F | - | -0.25 |
|  | 28 (14/14) | 6.0 (6) | Phonemic | COWAT/F, A, S | English | F | - | 0.35 |
|  | 28 (14/14) | 6.0 (6) | Semantic | Animals, Fruits/vegetables/food, Action Verbs | English | F | - | 1.11 |
|  | 31 (17/14) | 7.0 (7) | Phonemic | COWAT/F, A, S | English | F | - | -0.20 |
|  | 31 (17/14) | 7.0 (7) | Semantic | Animals, Fruits/vegetables/food, Action Verbs | English | F | - | 0.86 |
|  | 29 (13/16) | 8.0 (8) | Phonemic | COWAT/F, A, S | English | F | - | -0.25 |
|  | 29 (13/16) | 8.0 (8) | Semantic | Animals, Fruits/vegetables/food, Action Verbs | English | F | - | -0.45 |
|  | 27 (12/15) | 9.0 (9) | Phonemic | COWAT/F, A, S | English | F | - | -0.49 |
|  | 27 (12/15) | 9.0 (9) | Semantic | Animals, Fruits/vegetables/food, Action Verbs | English | F | - | 0.09 |
|  | 28 (17/11) | 10.0 (10) | Phonemic | COWAT/F, A, S | English | F | - | 0.13 |
|  | 28 (17/11) | 10.0 (10) | Semantic | Animals, Fruits/vegetables/food, Action Verbs | English | F | - | 0.04 |
|  | 24 (14/10) | 11.0 (11) | Phonemic | COWAT/F, A, S | English | F | - | 0.29 |
|  | 24 (14/10) | 11.0 (11) | Semantic | Animals, Fruits/vegetables/food, Action Verbs | English | F | - | 0.35 |
|  | 23 (11/12) | 12.0 (12) | Phonemic | COWAT/F, A, S | English | F | - | 0.21 |
|  | 23 (11/12) | 12.0 (12) | Semantic | Animals, Fruits/vegetables/food, Action Verbs | English | F | - | 0.26 |
| [Vakil and Blachstein (1997)](#_ENREF_237)^a^ | 117 (57/60) | 24.5 (20-29) | Recall | RAVLT | Hebrew | M | F | 0.32 |
|  | 117 (57/60) | 24.5 (20-29) | Recognition | RAVLT | Hebrew | M | F | -0.86 |
|  | 63 (39/24) | 33.2 (30-39) | Recall | RAVLT | Hebrew | M | F | 0.43 |
|  | 63 (39/24) | 33.2 (30-39) | Recognition | RAVLT | Hebrew | M | F | 0.84 |
|  | 86 (42/44) | 44.9 (40-49) | Recall | RAVLT | Hebrew | M | F | 0.15 |
|  | 86 (42/44) | 44.9 (40-49) | Recognition | RAVLT | Hebrew | M | F | -0.13 |
|  | 55 (27/28) | 53.6 (50-59) | Recall | RAVLT | Hebrew | M | F | 0.59 |
|  | 55 (27/28) | 53.6 (50-59) | Recognition | RAVLT | Hebrew | M | F | 0.42 |
|  | 115 (48/67) | 64.4 (60-69) | Recall | RAVLT | Hebrew | M | F | 0.52 |
|  | 115 (48/67) | 64.4 (60-69) | Recognition | RAVLT | Hebrew | M | F | 0.03 |
|  | 92 (44/48) | 76.6 (70-91) | Recall | RAVLT | Hebrew | M | F | -0.02 |
|  | 92 (44/48) | 76.6 (70-91) | Recognition | RAVLT | Hebrew | M | F | -0.06 |
| [van Hooren et al. (2007)](#_ENREF_239)^a^ | 578 (292/286) | 72.5 (64-81) | Recall | Visual Verbal Learning Test | Dutch | F | F | 0.50 |
|  | 578 (292/286) | 72.5 (64-81) | Semantic | Animals | Dutch | F | F | -0.07 |
| [Vannorsdall (2006)](#_ENREF_240)^p^ | 230 (111/119) | 58.8 (21-96) | Recall | Hopkins Verbal Learning Test, Logical Memory II compound score | English | F | - | 0.32 |
| [Wagner (1980)](#_ENREF_245)^p^ | 383 (192/191) | 6.0 (3-9) | Semantic | Animals, things to eat, wear, and ride | English | F | - | -0.01 |
| [Walburn (2014)](#_ENREF_247)^m^ | 53 (17/36) | 22.9 (19-40) | Recall | Generic word list | English | F | - | -0.54 |
| [Weiss et al. (2003)](#_ENREF_253)^a^ | 97 (46/51) | 25.1 | Phonemic | Starting letters: B, A, S | German | F | F | 0.45 |
|  | 97 (46/51) | 25.1 | Recognition | Warrinton Recognition Memory Battery | German | F | F | 0.32 |
|  | 97 (46/51) | 25.1 | Semantic | Animals, Fruits/vegetables/food, supermarket | German | F | F | 0.24 |
| [Weiss et al. (2006)](#_ENREF_254) | 80 (40/40) | 25.2 (16-34) | Phonemic | COWAT/F, A, S | German | F | F | 0.51 |
|  | 80 (40/40) | 25.2 (16-34) | Semantic | Animals | German | F | F | 0.05 |
| [Westcott (1969)](#_ENREF_255)^p^ | 90 (43/47) | 13 (Pupils grade 4. 8. 12) | Recall | Generic word list | English | F | - | 0.21 |
| [Wilkosc et al. (2016)](#_ENREF_256)^a^ | 152 (76/76) | 38.4 (18-60) | Recall total | RAVLT | Polish | F | M | 0.54 |
| [Willis (1997)](#_ENREF_257)^p^ | 30 (15/15) | 5.0 (5) | Semantic | Animals, Fruits/vegetables/food | English | F | - | 0.41 |
|  | 30 (15/15) | 6.0 (6) | Semantic | Animals, Fruits/vegetables/food | English | F | - | 0.21 |
| [Wolkenberg (1999)](#_ENREF_259)^p^ | 160 (72/88) | 20.1 (18-35) | Phonemic | COWAT/F, A, S | English | M | - | -0.03 |
|  | 160 (72/88) | 20.1 (18-35) | Semantic | Animals | English | M | - | -0.09 |
| [Wulandari and Hendrawan (2020)](#_ENREF_262)^a^ | 41 (19/22) | 20.5 (18-23) | Phonemic | Starting letters: K, R, W | Indonesian | F | M | 0.34 |
| [Xu (2018)](#_ENREF_263)^p^ | 12410 (5812/6598) | 62.5 (50+) | Recall | 10 Word Learning Test | Chinese | F | - | -0.04 |
|  | 12410 (5812/6598) | 62.5 (50+) | Semantic | Animals | Chinese | F | - | -0.22 |
|  | 6244 (3180/3064) | 61.2 (50+) | Recall | 10 Word Learning Test | India (not further specified) | F | - | -0.17 |
|  | 6244 (3180/3064) | 61.2 (50+) | Semantic | Animals | India (not further specified) | F | - | -0.33 |
| [Yeudall et al. (1986)](#_ENREF_265)^a^ | 225 (127/98) | 27.5 (15-40) | Phonemic | COWAT/F, A, S | English | F | M | 0.38 |
| [Yi (2007)](#_ENREF_266)^p^ | 210 (107/103) | 74.2 (66-89) | Phonemic | COWAT/F, A, S | English | F | - | -0.11 |
|  | 203 (103/100) | 74.2 (66-89) | Recall | CVLT | English | F | - | -0.57 |
|  | 211 (108/103) | 74.2 (66-89) | Recall | Logical Memory II from Wechsler Memory Scale | English | F | - | -0.09 |
|  | 203 (103/100) | 74.2 (66-89) | Recognition | CVLT | English | F | - | -0.60 |
|  | 211 (108/103) | 74.2 (66-89) | Recognition | Logical Memory Recognition from Wechsler Memory Scale | English | F | - | -0.18 |
|  | 210(107/103) | 74.2 (66-89) | Semantic | Animals | English | F | - | 0.06 |
| [Yonker et al. (2003)](#_ENREF_267)^a^ | 36 (18/18) | 60.7 (55>) | Phonemic | Starting letter: A, 5 letter words starting with M, Professions starting with B | Swedish | F | F | 0.33 |
|  | 36 (18/18) | 60.7 (55>) | Recall | Composite of word recall, performed activities, newly acquired facts | Swedish | F | F | 0.82 |
|  | 36 (18/18) | 60.7 (55>) | Recognition | Generic word list | Swedish | F | F | 0.36 |
| [Young (2002)](#_ENREF_268)^p^ | 57 (28/29) | 51.5 (18-85) | Phonemic | COWAT/F, A, S | English | F | - | 0.65 |
| [Zhu (2015)](#_ENREF_271)^p^ | 1619 (585/1034), Black Americans | 70.1 (45+) | Phonemic | Starting letter: F | English | F | - | 0.02 |
|  | 1932 (702/1230), Black Americans | 70.1 (45+) | Recall | 10 Word Learning Test from CERAD battery | English | F | - | 0.37 |
|  | 2000 (717/1283), Black Americans | 70.1 (45+) | Semantic | Animals | English | F | - | -0.20 |
|  | 3858 (1857/2001), White Americans | 70.1 (45+) | Phonemic | Starting letter: F | English | F | - | 0.15 |
|  | 4430 (2166/2264), White Americans | 70.1 (45+) | Recall | 10 Word Learning Test from CERAD battery | English | F | - | 0.55 |
|  | 4565 (2224/2341), White Americans | 70.1 (45+) | Semantic | Animals | English | F | - | 0.02 |
| [Zoccoli (2005)](#_ENREF_272)^m^ | 72 (28/44) | 19.1 (17-23) | Phonemic | Starting letters: F, A, S, W, C, P | English | F | - | -0.24 |
|  | 72 (28/44) | 19.1 (17-23) | Semantic | Animals, fruits/vegetables/food, occupation, musical instruments, chemical elements | English | F | - | -0.07 |

*Notes*: M = male, F = female; ^a^ = article (from Pubmed, ISI, PychInfo); ^p^ = PhD Thesis (ProQuest); ^m^ = Master’s Thesis (ProQuest); CVLT = California Verbal Learning Test; RAVLT = Rey Auditory Verbal Learning Test, CERAD = Consortium to Establish a Registry for Alzheimer's Disease; ImPACT= Immediate Post-Concussion Assessment and Cognitive Testing; PVLT = Philadelphia Verbal Learning Test; (positive *d* values indicate a female advantage).

# Figure S1

*Funnel plots for measures of verbal ability*


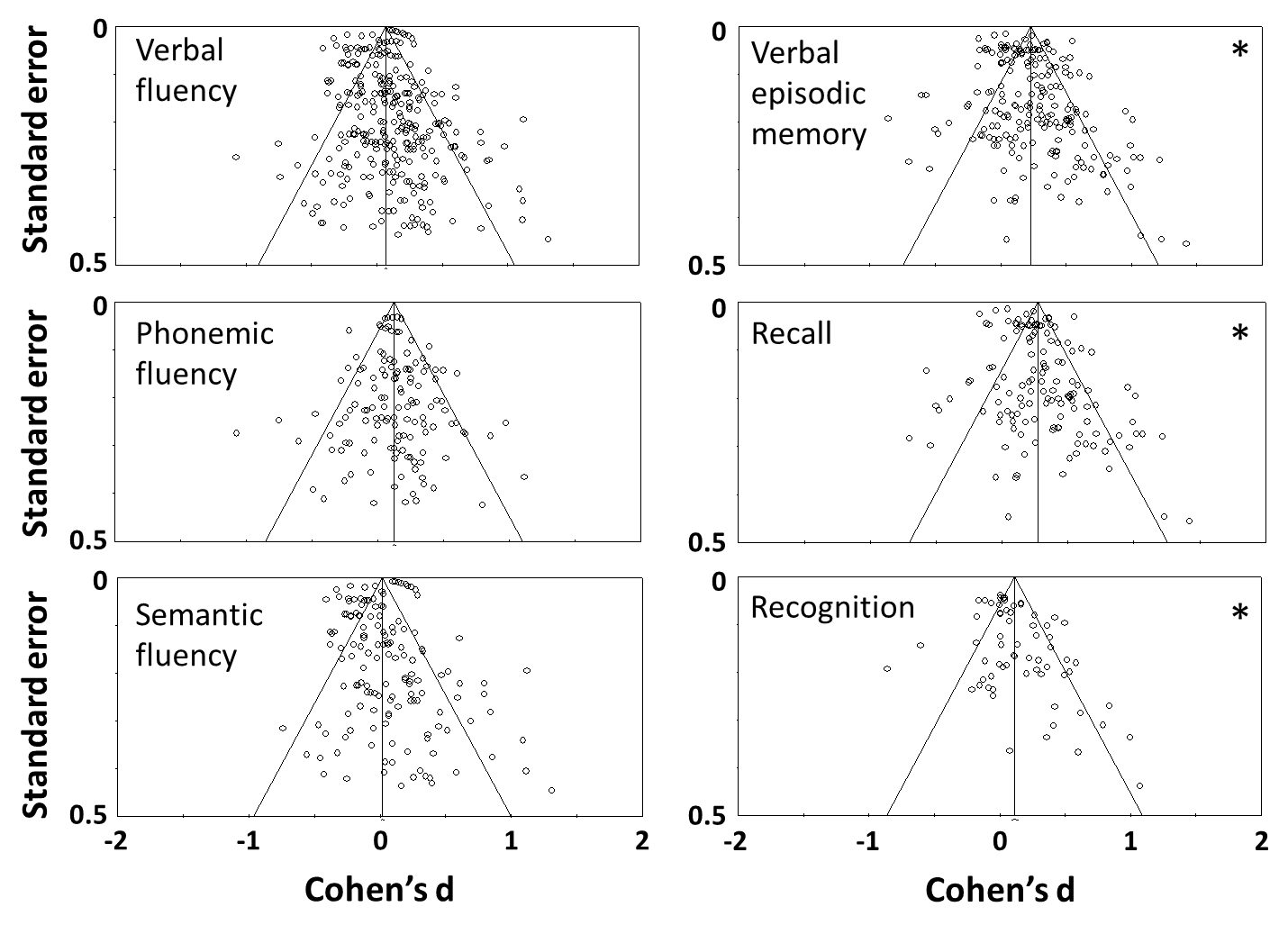


*Note*. Funnel plots are based on assuming perfect independence between multiple measures from the same (sub-)sample. * denotes a significant Egger’s intercept implying a small sample bias.

# Figure S2

*Effect of publication year*


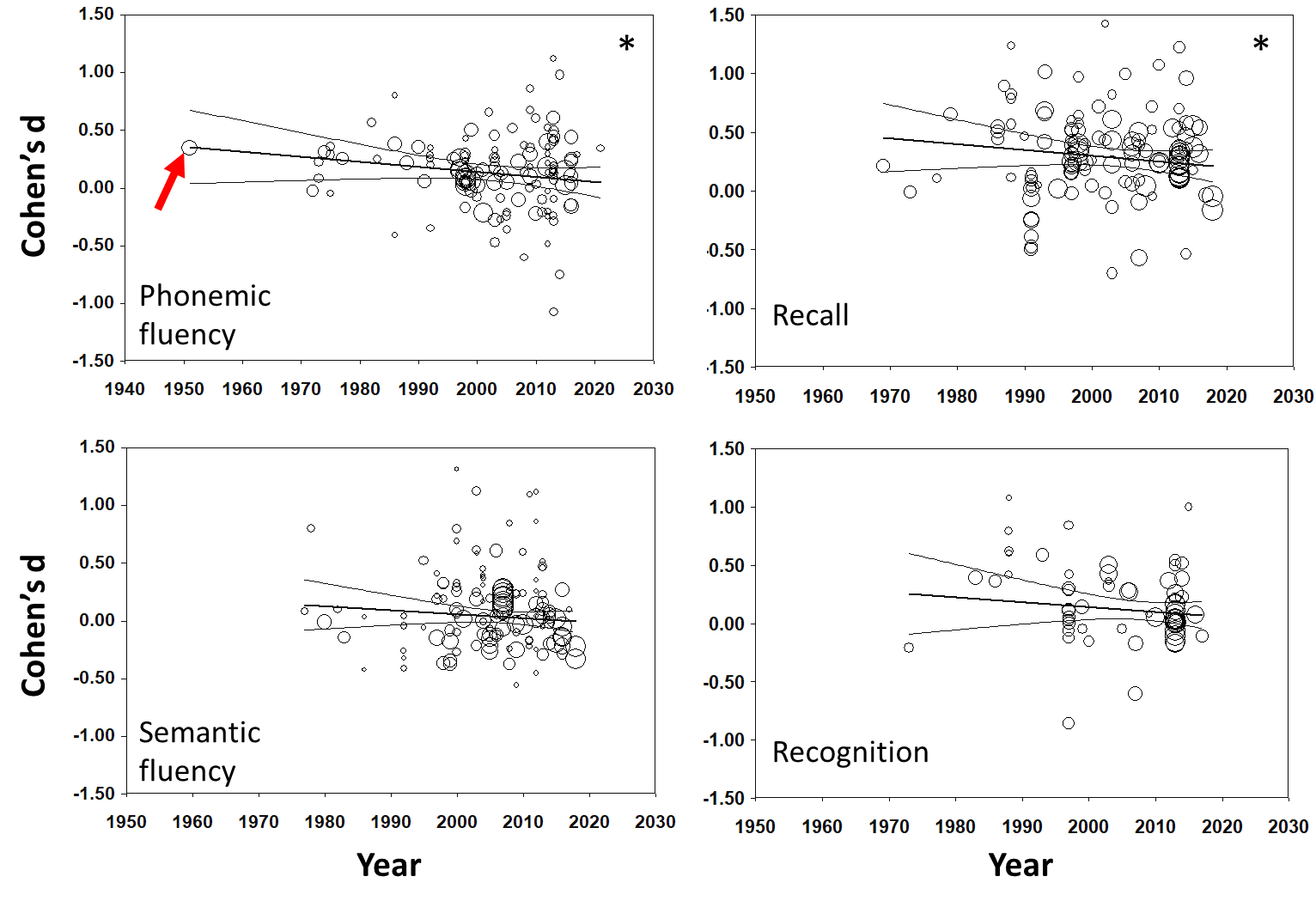


*Note*. Regression lines are accompanied by upper and lower lines representing 95% confidence bands. * denotes significant effect (*p*<.05). If Elias (1951) is removed (see red arrow), the effect is no longer significant for phonemic fluency. The figure is based on assuming perfect independence of multiple measures from the same (sub-)sample.

# Figure S3

*Effect of Age mean*


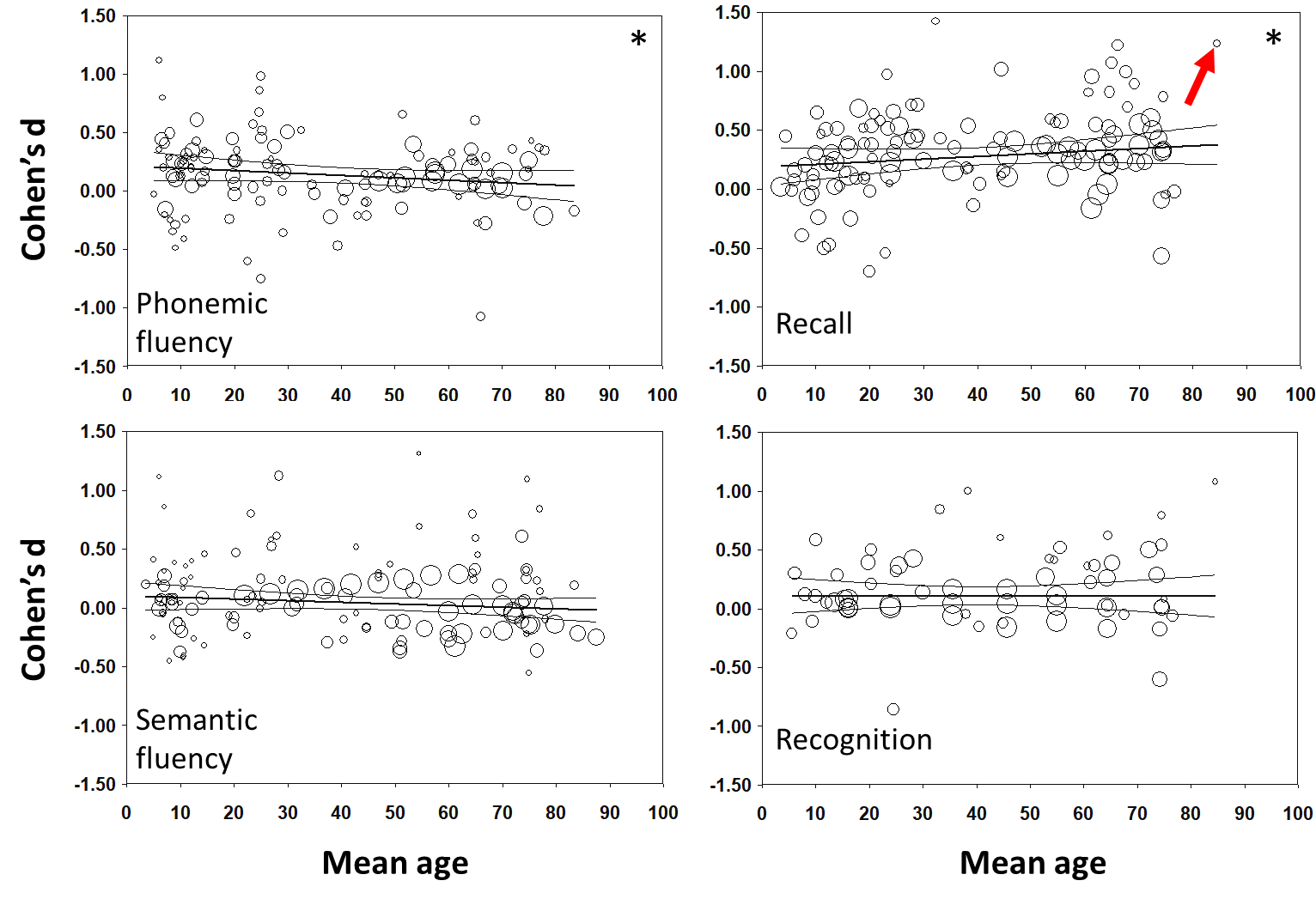


*Note*. Regression lines are in the middle, upper and lower lines represent confidence bands. * denotes significant effect. If Bleecker et al. (1988) is removed (see red arrow), the effect is no longer significant for recall. Figures are based on assuming perfect independence of multiple measures from the same (sub-)sample.

# Figure S4

*Gender of last author effect*


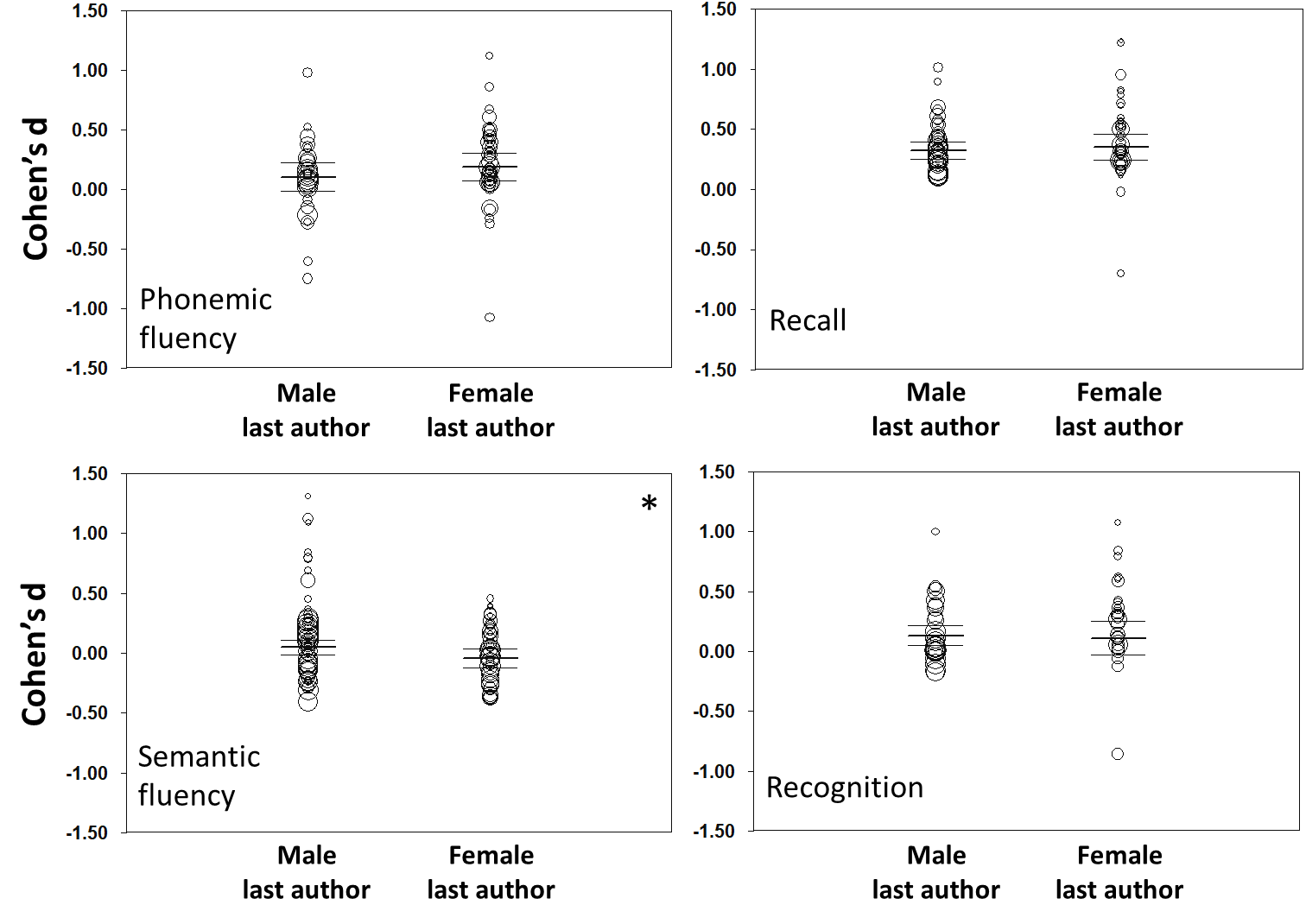


*Note*. * denotes significant difference between female and male last authors. Central lines represent means of the respective category, upper and lower lines are confidence intervals. Figures are based on assuming perfect independence between multiple measures from the same (sub-) sample.
